# Contemporary ancestor? Adaptive divergence from standing genetic variation in Pacific marine threespine stickleback

**DOI:** 10.1186/s12862-018-1228-8

**Published:** 2018-07-18

**Authors:** Matthew R. J. Morris, Ella Bowles, Brandon E. Allen, Heather A. Jamniczky, Sean M. Rogers

**Affiliations:** 1 Department of Biology, Ambrose University, 150 Ambrose Circle SW, Calgary, AB T3H 0L5 Canada; 2 Department of Biological Sciences, University of Calgary, 2500 University Dr. NW, Calgary, AB T2N 1N4 Canada; 3 McCaig Institute for Bone and Joint Health, Department of Cell Biology & Anatomy, University of Calgary, 3330 Hospital Dr. NW, Calgary, AB T2N 4Z6 Canada

**Keywords:** Evolutionary genomics, Adaptive radiation, Next-generation sequencing, Morphological evolution, Population structure, Natural selection

## Abstract

**Background:**

Populations that have repeatedly colonized novel environments are useful for studying the role of ecology in adaptive divergence – particularly if some individuals persist in the ancestral habitat. Such “contemporary ancestors” can be used to demonstrate the effects of selection by comparing phenotypic and genetic divergence between the derived population and their extant ancestors. However, evolution and demography in these “contemporary ancestors” can complicate inferences about the source (standing genetic variation, de novo mutation) and pace of adaptive divergence. Marine threespine stickleback (*Gasterosteus aculeatus*) have colonized freshwater environments along the Pacific coast of North America, but have also persisted in the marine environment. To what extent are marine stickleback good proxies of the ancestral condition?

**Results:**

We sequenced > 5800 variant loci in over 250 marine stickleback from eight locations extending from Alaska to California, and phenotyped them for platedness and body shape. Pairwise F_ST_ varied from 0.02 to 0.18. Stickleback were divided into five genetic clusters, with a single cluster comprising stickleback from Washington to Alaska. Plate number, *Eda*, body shape, and candidate loci showed evidence of being under selection in the marine environment. Comparisons to a freshwater population demonstrated that candidate loci for freshwater adaptation varied depending on the choice of marine populations.

**Conclusions:**

Marine stickleback are structured into phenotypically and genetically distinct populations that have been evolving as freshwater stickleback evolved. This variation complicates their usefulness as proxies of the ancestors of freshwater populations. Lessons from stickleback may be applied to other “contemporary ancestor”-derived population studies.

**Electronic supplementary material:**

The online version of this article (10.1186/s12862-018-1228-8) contains supplementary material, which is available to authorized users.

## Background

The ecological theory of adaptive divergence predicts that populations diverge phenotypically and genetically if they reside in distinct environments [1, 2], potentially resulting in speciation. Some of the most striking examples of adaptive divergence come from species in which contemporary populations persist in environments putatively occupied by ancestral populations (e.g. [3–11]). To the extent that such populations have not undergone evolution, contemporary descendants of populations in ancestral environments can be used as a proxy for the ancestor, with phenotypic and genetic differences between these “contemporary ancestors” and “derived” populations being used to infer the direction, source, and pace of adaptation to derived environments. However, recent changes in the ancestral environment may stimulate evolutionary responses in the contemporary populations that inhabit it, complicating their utility as a proxy.

Standing genetic variation (SGV), defined as the variety of alleles segregating in a population [12, 13], is expected to play an important role in parallel evolution. In particular, SGV permits rapid adaptation compared to de novo mutation, and increases the likelihood that the same beneficial allele will be present in different derived populations [14–16]. The role of SGV in adaptive divergence is readily measurable: if an allele fixed in the derived population is present in the contemporary ancestor at low frequencies, it likely contributed to adaptation [13]. However, the inference that an allele present in the contemporary ancestral population resulted in adaptation via SGV requires three assumptions. (1) The subset of individuals that originally colonized the derived environment must have contained the rare adaptive allele at some frequency; otherwise it arose from de novo mutation or subsequent gene flow. (2) The contemporary ancestor has undergone little evolution, including gene flow from the derived population (e.g., [17]). (3) The ancestral population has to have been properly characterized (i.e., population structure and allele frequencies associated with SGV). If there are multiple potential ancestral populations, each with a different pool of SGV, inference about the source and pace of evolution in the derived population will vary depending on which putative ancestral population is investigated (e.g. [12]). These assumptions must be verified to characterize accurately the role of SGV during population divergence.

Threespine stickleback (*Gasterosteus aculeatus*) provide perhaps the best documented examples of adaptation from SGV. Marine threespine stickleback occur widely in the northern hemisphere, including along the Pacific coast of North America from Alaska south to southcentral California. Across the north Pacific coast, much freshwater habitat formed recently (~ 10,000–20,000 years ago) in association with isostatic rebound following glacial retreat. The subsequent colonization of this habitat by stickleback allows tests of the significance of de novo mutation and SGV for adaptation (e.g. [17–19]). For instance, marine stickleback bodies are often covered by > 29 bony lateral plates, but fewer plates (0–10) have evolved in parallel in freshwater populations through selection on a rare marine allele [18]. Despite numerous studies indicating the role of SGV at either a single locus for platedness (*Ectodysplasin –* hereafter *Eda*) or for multiple loci with unknown phenotypic effects [20–22], assumptions about the appropriateness of considering extant marine sticklebacks as representative of the ancestors of freshwater populations remains untested. Despite evident genetic variation in threespine stickleback among geographic clades [23–26], marine stickleback on the eastern Pacific are largely assumed to constitute a single population (e.g. [27–31]). This assumption is justified by the absence of barriers to gene flow in the marine environment [27], the migratory capacity of marine stickleback [32], the relative “evolutionary stasis” of marine stickleback inferred from the fossil record [28, 29], and the low marine population structure reported from several local studies ([20, 33], but see [34]). Nevertheless, substantial evidence indicates local adaptation even in highly migratory marine fishes [35–37], and indeed in Baltic Sea threespine stickleback [38–40]. If Pacific threespine stickleback constitute a single population, their large population size should limit the effects of genetic drift and high gene flow should offset local adaptation [41]. Given these conditions, marine stickleback should not exhibit local differentiation that would generate regional differences in the initial colonists of freshwater lakes and streams. SGV should be the same along the Pacific coast – and all freshwater populations could have evolved from the same initial pool of marine SGV facilitating parallel adaptation. These assumptions require formal testing to elucidate the role of SGV during adaptive divergence.

In this study, we consider phenotypic and genotypic variation of > 200 marine threespine stickleback from eight locations from California to Alaska to test hypotheses about the genetic structure of marine stickleback and its evolutionary consequences. Based on variation in plate phenotypes and genotypes associated with SGV at *Eda*, three-dimensional body morphology from micro-computed tomography (μCT) scans quantified using geometric morphometrics, and Genotype-by-Sequencing [42], we assess whether marine stickleback constitute a single population. By doing so, we test assumptions about the distribution of SGV in “contemporary ancestors” of freshwater stickleback. Additionally, we test predictions regarding the influence of SGV on the source of adaptation based on genomic sequences for a freshwater population from British Columbia. We specifically test the following null predictions: (1) Marine stickleback populations will not vary in the frequency and content (e.g. private alleles) of SGV. (2) Similarly, marine stickleback will not exhibit genetic population structure, which would otherwise influence the SGV regionally available for selection. (3) Marine populations will exhibit no phenotypic divergence in body shape or platedness [e.g. 18]. (4) If differences in SGV among marine populations occurs, genetic variation will show no evidence of having been shaped by natural selection. (5) If population structure in marine stickleback occurs, geographic proximity to a freshwater population will determine the extent of genetic divergence between marine and freshwater stickleback. (6) Differences in SGV among marine populations, if they occur, will not affect the candidate loci identified as contributing to adaptation in freshwater stickleback.

## Methods

**Fig. 1:**
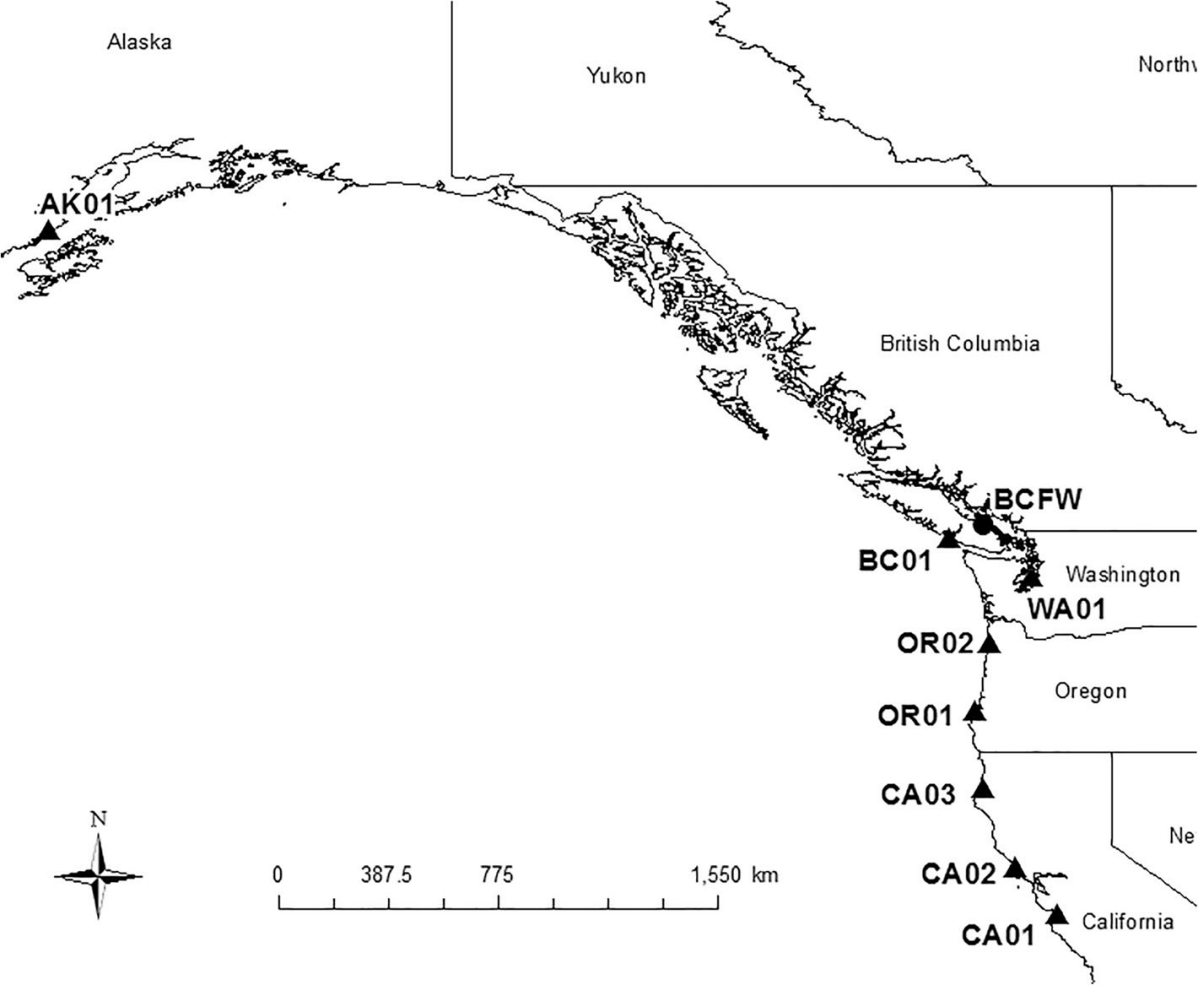
Map of sampling localities. See Table 1 for code designations. Marine sites = triangles, freshwater site = circle

Threespine stickleback (*n* = 383, Table 1) were collected with minnow traps or seines during the summers of 2010 (Brannen Lake, British Columbia, hereafter BCFW), 2012 (Alaska) and 2013 (all other localities). Sampling locations extended along a 21.8 degree latitudinal spread (Table 1, Fig. 1), from California (south to north, CA01, CA02, CA03), through Oregon (OR01, OR02), the Puget Sound area of Washington (WA01), Vancouver Island (BC01) and Alaska (AK01). Locations varied in terms of benthos, freshwater input, and protection – for instance, CA01 fish were sampled in a slough with freshwater input determined by precipitation, while OR02 were collected near a tidal gate close to the mouth of a river. Other marine species were collected alongside stickleback, such as bay pipefish (*Syngnathus leptorhyncus*) or smelt (*Atherinops/Atherinopsis* sp.). Adults were captured in all localities with the exception of WA01, while OR01 contained a range of age classes. Stickleback were euthanized using buffered tricaine methanesulfonate (MS-222) or Eugenol (clove oil) and preserved in 70% ethanol. Fin clips were preserved in 95% ethanol for later sequencing. All collections were conducted in accordance with CCAC guidelines (AUP AC13–0040) and state/provincial/national collection and import permits.

**Table 1:** Information about the sampling of threespine stickleback, and the number used for various analyses

Site name	Water body	State/ Province	Latitude	Longitude	N	N > 30 mm	N morph	N pstacks
CA01	Elkhorn Slough	California	36°49’45N	121°44’07W	35	35	35	29
CA02	Doran Park	California	38°18’52N	123°01’55W	50	48	48	28
CA03	Arcata Marsh	California	40°51’23N	124°05’24W	50	46	44	28
OR01	South Slough	Oregon	43°17’35N	124°19’26W	51	20	20	29
OR02	Tillamook Bay	Oregon	45°28’52N	123°53’49W	50	50	48	30
WA01	Little Clam Bay	Washington	47°34’32N	122°32’43W	50	0	0	25
BC01	Bamfield Inlet	British Columbia	48°49’55N	125°08’17W	51	51	48	31
AK01	Swikshak Lagoon	Alaska	58°37’14N	153°44’44W	31	31	29	24
BCFW	Brannen Lake	British Columbia	49°12’54N	124°03’16W	15	NA	NA	15

N represents the total number captured. N > 30 mm is the total number used for plate counts. N morph is the number > 30 mm that were used for 3-D morphometrics. N pstacks is the number of individuals included in the sequencing run that passed the *process_radtags* filter (see text)

Sex was identified using primers developed by [43] that amplify sex-specific alleles at the *idh* locus. Alleles were visualized in a 2% agarose gel for 367 individuals.

### Library preparation and analysis

Reduced representation DNA sequencing was used to generate Single Nucleotide Polymorphisms (SNPs) in order to assess population structure and adaptive divergence. Two hundred nanograms total genomic DNA was extracted per fish in January 2016 using Qiagen DNeasy Blood and Tissue kits (*n* = 265) and digested with EcoRI and MseI restriction enzymes (chosen after in silico digestion [44]). Thirty to thirty-five individuals were included from each marine location, and 15 from BCFW. After digestion-ligation, fish were pooled into groups of nine. Cleanup and size selection were performed simultaneously using SPRI beads (Beckman Coulter), at a bead ratio of 0.8× and 0.61× for left and right-side cleanup, respectively. This left a fragment range between 250 and 600 bp. Pooled samples were divided into three technical replicates to ameliorate stochastic differences during PCR, and were amplified. Replicates were pooled and left-side cleaned using SPRI beads. Pooled samples were quantified using a 2200 TapeStation (Agilent) and Qubit (Thermofisher) dsDNA high sensitivity assay. Equal volumes of each 2 nM pooled sample were pooled to make the final library. Library preparation followed the Illumina protocols for the Illumina NextSeq 500 Mid-Output kits with version 2 chemistry. Two sequencing runs were completed on the Illumina Next Seq 500 using 150 cycles and different final library concentrations - the first at 1.8 pM final concentration, the second at 1.1 pM final concentration. A 20% PhiX spike-in was used for both to compensate for the low diversity nature of the library. Results from the two sequencing runs were merged.

Sequenced reads were cleaned and processed using *Stacks v.1.35* [45, 46]. Reads were de-multiplexed and cleaned using *process_radtags*, rescuing barcodes if the correction of a single sequencing error made them identifiable. *GSnap* [47] was used to align reads to the stickleback reference genome (Ensembl release 72 [48]), allowing for five mismatches with soft masking disabled. SNP calls were corrected in *rxstacks* using a bounded SNP model with an upper error rate of 0.1. Stringent filtering criteria were applied to the data, with slightly different filtering criteria used to address different questions. In order to determine population structure, each sampling site was assumed to constitute a distinct population. The filtering criteria for this “marine site” data set included: i) log likelihood threshold > − 60; ii) sequenced in more than 75% of individuals; iii) in 6 of 8 populations; iv) with minimum 4× coverage; v) minimum minor allele frequency of 2%, and vi) F_IS_ > − 0.3. Individuals were included if they retained > 10,000 RAD-loci after cleaning (*n* = 239, Table 1). After population structure was determined, *Adegent*-recognized clusters rather than sampling location were used in the second *Stacks* run. This “marine cluster” data set used the same filtering criteria as above, with the exception that the variant needed to be present in all clusters. Finally, a “marine-freshwater” data set was run, which treated each sampling site as a distinct population and additionally included a freshwater population (see below). In this case the variant needed to be present in all eight marine populations and in the freshwater population. For each data set, all population statistics except for F_ST_ were calculated using the *populations* module of *Stacks*.

### Population genetic structure

Pairwise global and per-locus F_ST_ were calculated using the Weir and Cockerham [49] adaptation implemented in *hierfstat v0.04–22* [50] in R [51]; pairwise global F_ST_ were tested for significance (> 0) using 999 permutations in GenoDive [52]. Discriminant Analysis of Principal Components (DAPC) [53] was used on the “marine site” data to assess population structure in the marine environment using *Adegenet v2.0.1* [54], as it has shown to perform better than *Structure* under a stepping-stone model of dispersal [53]. The optimal number of Principal Components (PCs) to retain was calculated using both xvalDAPC and a-scores, which gave similar answers. The optimal number of clusters was assigned based on the lowest Bayesian Information Criteria (BIC) score using *k*-means clustering. As several possible *k* clusters had similarly low BIC scores, analyses were run and compared using 3 to 8 clusters.

An analysis of molecular variance (AMOVA), implemented in *poppr v2.3.0* [55, 56] using the *Ade4* package [57], was used to determine the proportions of genetic variance among versus within sampling sites or *Adegenet*-recognized clusters. Missing values were replaced with the average frequency for a locus; ignoring missing values did not alter overall patterns. To explore the possibility of cryptic population structure, each sampling site was further analysed individually using *Stacks* and *Adegenet*.

The distance between each sampling site was measured as distance along the coast (km) using Google Maps. Distances were measured to or from the mouth of each bay. Neighbouring localities were separated by 242–479 km, except for BC01-AK01, which were separated by approximately 2500 km of coastline. The location of WA01 in Puget Sound resulted in all locations south of Washington being closer to BC01 than they were to WA01. Genetic distance was calculated using the pairwise global Weir and Cockerham F_ST_ measures from *hierfstat*. Geographic and genetic distance matrices were compared using a Mantel test from the *Adegenet* package with 999 replications to determine Isolation-by-Distance (IBD).

Population statistics were also estimated for the “marine cluster” data set in *Stacks* using the optimal *Adegenet*-identified clusters.

A phylogenetic network was calculated using SNPhylo [58] and visualized using FigTree v.1.4.3 [59]. The “marine sites” data set was used, but SNPhylo additionally filtered loci based on linkage disequilibrium. Individuals were colour-coded according to their recognized genetic cluster. A hierarchical clustering tree was additionally constructed using *BayPass v.2.1* [60].

### Platedness

Plate variation among populations was assessed using plate number and *Eda* genotype. Adult stickleback (i.e. fish > 30 mm standard length (SL)) (*n* = 281, Table 1, Additional file 1: Table S2) were stained in Alizarin red. Plate number, including keel, was counted on both sides of the body and summed. Low-plated individuals without keel (LPNK) were defined as individuals with < 20 anterior plates. Partially-plated keeled (PPK) fish had 21–59 plates, including at least one plate at the caudal keel. Fully-plated keeled (FPK) fish had ≥ 60 plates. Additionally, some low-plated fish had a keel (LPK) and were defined as having < 20 anterior plates plus additional plates at the caudal keel. Partially plated stickleback that lacked a keel (PPNK) had > 20 anterior plates but had no plates at the caudal keel. Individuals were also genotyped at the *Stn382* locus [18] as this microsatellite is linked to an indel in intron 1 of the *Eda* gene, yielding a 218 bp “fully-plated” allele (C) or a 158 bp “low-plated” allele (L) [61]. Genotyping followed the protocol of [43]. Individuals were genotyped as LL (homozygous for the low-plated allele), CL (heterozygous), or CC (homozygous for the fully-plated allele). This approach allowed juveniles (< 30 mm SL) to be included in the analysis (total *n* = 361), and provided genetic information at a locus with known adaptive significance that was not recovered from sequencing. Hardy-Weinberg equilibrium (HWE) was assessed for *Stn382* for each marine site using a goodness-of-fit Chi-squared test.

### Morphometrics

**Fig. 2:**
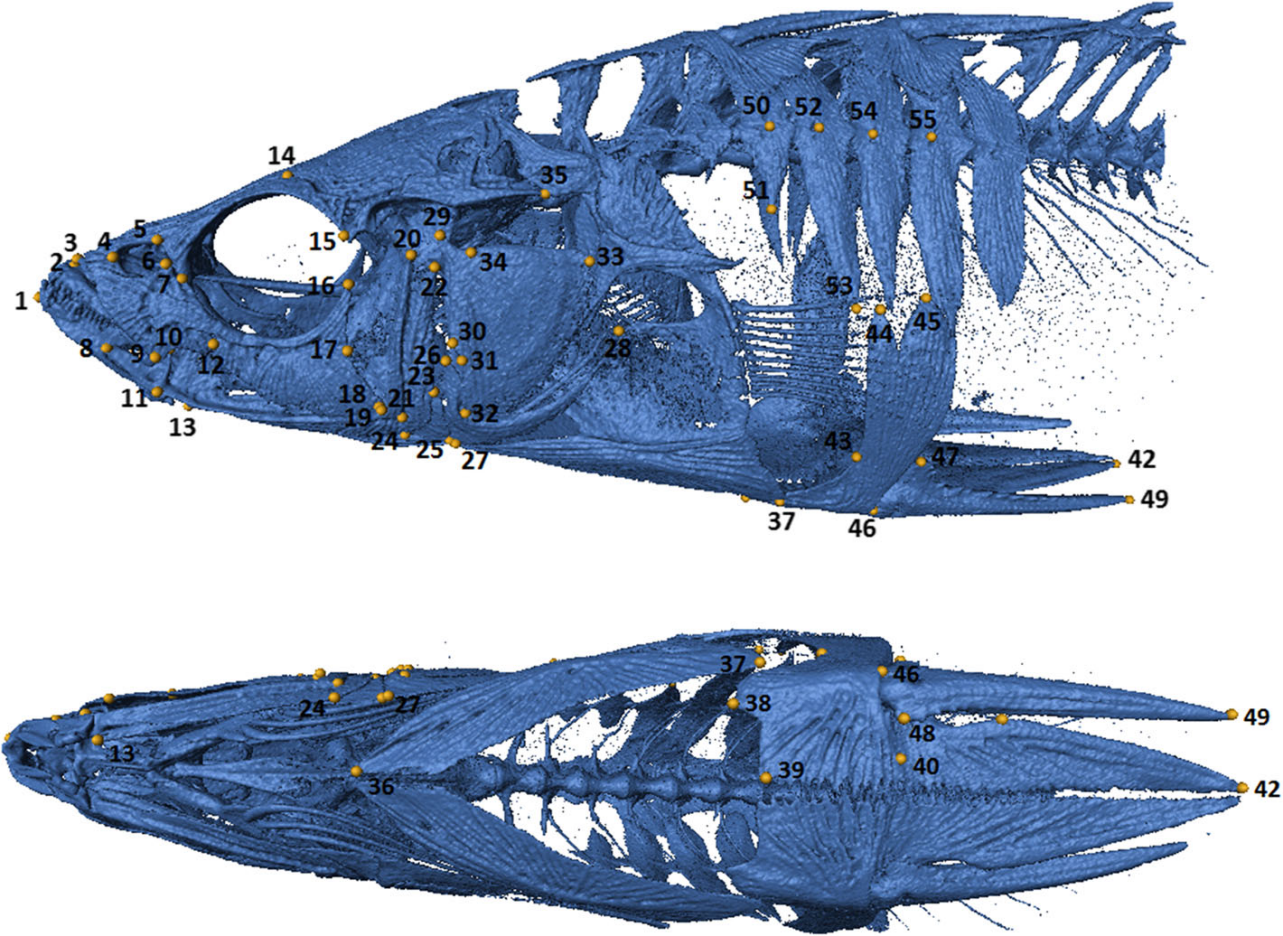
Position of 55 landmarks used for the morphometric analysis. See Table S1 for identity of landmarks

Phenotypic variation among populations was further assessed using morphometric analysis. Stickleback > 30 mm SL that had been preserved with relatively little bending (*n* = 272) were straightened, and spines and fins held flat against the body using plastic wrap. μCT scanning at a resolution of 20 μm was conducted in a standardized fashion for all individuals using a Scanco μCT35 instrument (Scanco AG). Three-dimensional images were generated from the anterior point of the premaxilla to the posterior tip of the pelvic spine, using standardized isosurface thresholds in *Amira 5.4* (FEI Visualization Sciences Group). Fifty-five landmarks were plotted on the left side of each fish (Additional file 1: Table S1, Fig. 2) and raw landmark scores were exported to *MorphoJ v1.06a* [62] for further analyses. A prior study had removed the operculum on the left side of all AK01 stickleback, so landmarks were plotted on their right sides. Data were first transformed to remove differences associated with isometric scaling, rotation and translation using Procrustes superimposition. Residuals from a within-marine site multivariate regression on centroid size were estimated and used in all subsequent analyses. Principal Components Analysis (PCA) determined the major axes of phenotypic variation. Canonical Variate Analysis (CVA) was used to determine Procrustes distances using sex and marine site as categorical variables, although BC01 had only one female and OR01 included one individual of unknown sex. The significance of Procrustes distances among pairwise comparisons of marine site-sex combinations was determined based on 10,000 permutations and a corrected α of 0.0005. Discriminant Function Analysis (DFA) was used to determine the likelihood that individuals could be reassigned to their site of origin, given their phenotypes. For this analysis the effect of sex on reassignment success was not assessed.

### Selection on phenotypic variation

Patterns of phenotypic variation among populations may be the result of genetic drift, natural selection, or phenotypic plasticity. One way to rule out neutral evolutionary processes is to compare estimates of phenotypic divergence (P_ST_) with neutral expectations based in part on observed neutral genetic differentiation. Observed phenotypic divergence was estimated as:

$$ {\mathrm{P}}_{\mathrm{ST}}\kern0.5em =\kern0.5em {\upsigma^2}_{\mathrm{B}}/\left({\upsigma^2}_{\mathrm{B}}\kern0.5em +\kern0.5em 2{\upsigma^2}_{\mathrm{W}}\right) $$

where σ^2^_B_ and σ^2^_W_ were the between- and within-population components of variance, respectively, for plate count and the first four PCs from the morphometric analysis (as per [63, 64]). Variance components were estimated for all marine sampling sites together (global P_ST_) and pairwise using *lme4* [65], with population as a random effect. Genetic divergence at the *Stn382* locus for *Eda* (F_STQ_) was estimated using the Weir and Cockerham method in *Genepop V4* [66]. Neutral genetic divergence (F_ST_) was estimated in *hierfstat* using non-genic SNPs identified from our data set using Biomart [67]. Non-genic SNPs may still be linked to loci under selection, so this approach provides a conservative estimate of neutrality.

Selection was inferred based on two methods. The first assessed the association between P_ST_-F_ST_ and F_STQ_-F_ST_ using Mantel tests. This measure is based on the expectation that phenotypic or QTL divergence will be uncorrelated with neutral genetic divergence – by extension implicating selection to explain such patterns. The second test involved Whitlock and Guillaume’s [68] method using the R-code from [69]. In brief, the expected between-population variance component for a neutral phenotype was estimated by using observed non-genic F_ST_ and observed within-population variance component for the phenotype:

$$ {\upsigma^2}_{\mathrm{B}}\kern0.5em =\kern0.5em 2{\mathrm{F}}_{\mathrm{ST}}\kern0.5em {\upsigma}^2\mathrm{w}/\left(1\hbox{-} {\mathrm{F}}_{\mathrm{ST}}\right) $$

As per [69], the distribution of neutral σ^2^_B_ was estimated by generating a χ^2^ distribution with six degrees of freedom (one less the number of sampling sites excluding Washington), and multiplying a randomly drawn value from this distribution by σ^2^_B_. From this new distribution expected neutral σ^2^_B_ were drawn 10,000 times and used to create a distribution of neutral P_ST_-F_ST_. The observed P_ST_-F_ST_ was then compared to this distribution and the quantile of the neutral distribution that lay beyond the observed value was used as the probability of the observed outcome in the absence of selection, *p*. Under the expectation of no selection, *p* is > 0; selection is evidenced if *p* = 0. F_STQ_-F_ST_ values was also compared to the neutral P_ST_-F_ST_, as per [40].

To quantify the relation between phenotype and *Stn382* genotype, a generalized linear model (GLM) was fit to the data in R, using the *glm* routine, as per [40], with plate number as the dependent variable, genotype as a fixed effect, and using a log-link function with a quasi-Poisson error distribution. Furthermore, a Mantel test was used to estimate the correlation between pairwise F_STQ_ and P_ST_ measures.

### Selection on genetic variation in the ocean

Under the assumption that marine stickleback populations have a shared history, the covariance matrix of population allele frequencies (Ω) was estimated in *BayPass* [60] using the “marine site” data. From this a hierarchical clustering tree [60] was generated, assuming no gene flow. A covariate-free genome scan was then performed to identify outlier loci putatively under selection, using per-locus measures of differentiation (XtX). The *simulate.baypass* function was used to estimate the posterior predictive distribution of XtX using a pseudo-observed data set (POD) [60]. Any loci in the “marine site” data set with XtX values above the POD-estimated threshold were scored as outlier loci potentially under selection. Genic outliers were identified using BioMart [67].

### Marine-freshwater genetic divergence

To assess the extent to which the choice of putative “contemporary ancestor” affected inference about adaptation to fresh water, pairwise F_ST_ was estimated for each marine-freshwater pair using *hierfstat*. In *BayPass*, covariance matrices, PODs and XtX thresholds were estimated for each marine-freshwater comparison. Outlier loci were examined to determine if the same outliers were being consistently recovered irrespective of the origin of the marine fish.

## Results

### Sex

A total of 205 males and 162 females were sampled. Sex bias was particularly striking in CA01 (26 M, 9 F), BC01 (47 M, 4 F), and AK01 (8 M, 23 F).

### Sequencing results

Over 192 million reads passed initial filters (Additional file 1: Table S3) in the “marine sites” data set. Two hundred eighty-two RAD-loci were excluded due to excess heterozygosity. Filtering minor alleles at a threshold of 2% reduced the number of retained loci by 32%. After filtering, between 230,010 (AK01) and 426,018 (OR01) loci were retained for each site sampled, generating between 1877 (AK01) and 5204 (OR01) SNPs (Table 2), for a total of 6655 variant loci.

**Table 2:** Full population genetic statistics for the filtered data set of marine stickleback

Variant loci												
Pop	Private	N	% Poly	P	Het_O_	H_O_	Het_E_	H_E_	π	F_IS_		
CA01	131	25.46	56.32	0.905	0.131	0.869	0.138	0.862	0.141	0.032		
CA02	103	25.02	69.39	0.901	0.141	0.859	0.149	0.851	0.153	0.041		
CA03	8	23.57	68.84	0.906	0.130	0.870	0.141	0.859	0.144	0.050		
OR01	17	25.57	80.42	0.897	0.145	0.856	0.158	0.842	0.162	0.064		
OR02	67	26.56	79.52	0.877	0.166	0.834	0.178	0.822	0.181	0.055		
WA01	4	21.74	65.57	0.915	0.119	0.881	0.130	0.871	0.133	0.049		
BC01	25	27.43	62.22	0.916	0.115	0.886	0.125	0.875	0.128	0.048		
AK01	1	20.00	57.09	0.927	0.103	0.897	0.111	0.889	0.114	0.048		
All sequenced loci												
Pop	Loci	Variant	SNP	% Poly	N	P	Het_O_	H_O_	Het_E_	H_E_	π	F_IS_
CA01	407925	6124	3449	0.846	25.9	0.9986	0.0020	0.9980	0.0021	0.9979	0.0021	0.0005
CA02	422173	6406	4445	1.053	25.4	0.9985	0.0021	0.9979	0.0023	0.9977	0.0023	0.0006
CA03	398661	5812	4001	1.004	23.9	0.9986	0.0019	0.9981	0.0021	0.9979	0.0021	0.0007
OR01	426018	6471	5204	1.222	26.0	0.9984	0.0022	0.9978	0.0024	0.9976	0.0025	0.0010
OR02	410111	6216	4943	1.205	26.9	0.9981	0.0025	0.9975	0.0027	0.9973	0.0027	0.0008
WA01	395684	5963	3910	0.988	21.9	0.9987	0.0018	0.9982	0.0020	0.9980	0.0020	0.0007
BC01	400271	5972	3716	0.928	27.7	0.9988	0.0017	0.9983	0.0019	0.9981	0.0019	0.0007
AK01	230010	3288	1877	0.816	20.2	0.9990	0.0015	0.9985	0.0016	0.9984	0.0016	0.0007

Private = Private alleles. N = Number of individuals used. P = Average major allele frequency. Het_O_ = Observed heterozygosity. Het_E_ = Expected heterozygosity. H_O_ = Observed homozygosity. H_E_ = Expected homozygosity. π = Nucleotide diversity. F_IS_ = Inbreeding coefficient. Loci = Average number of loci that were sequenced. Variant = Number of loci that were polymorphic in at least one marine site and sequenced in the site of interest. SNP = Number of Single Nucleotide Polymorphisms. % Poly = Proportion of variant loci that were polymorphic in the marine site of interest (top) or the proportion of sequenced loci that were polymorphic in the marine site of interest (bottom)

### Standing genetic variation

**Fig. 3:**
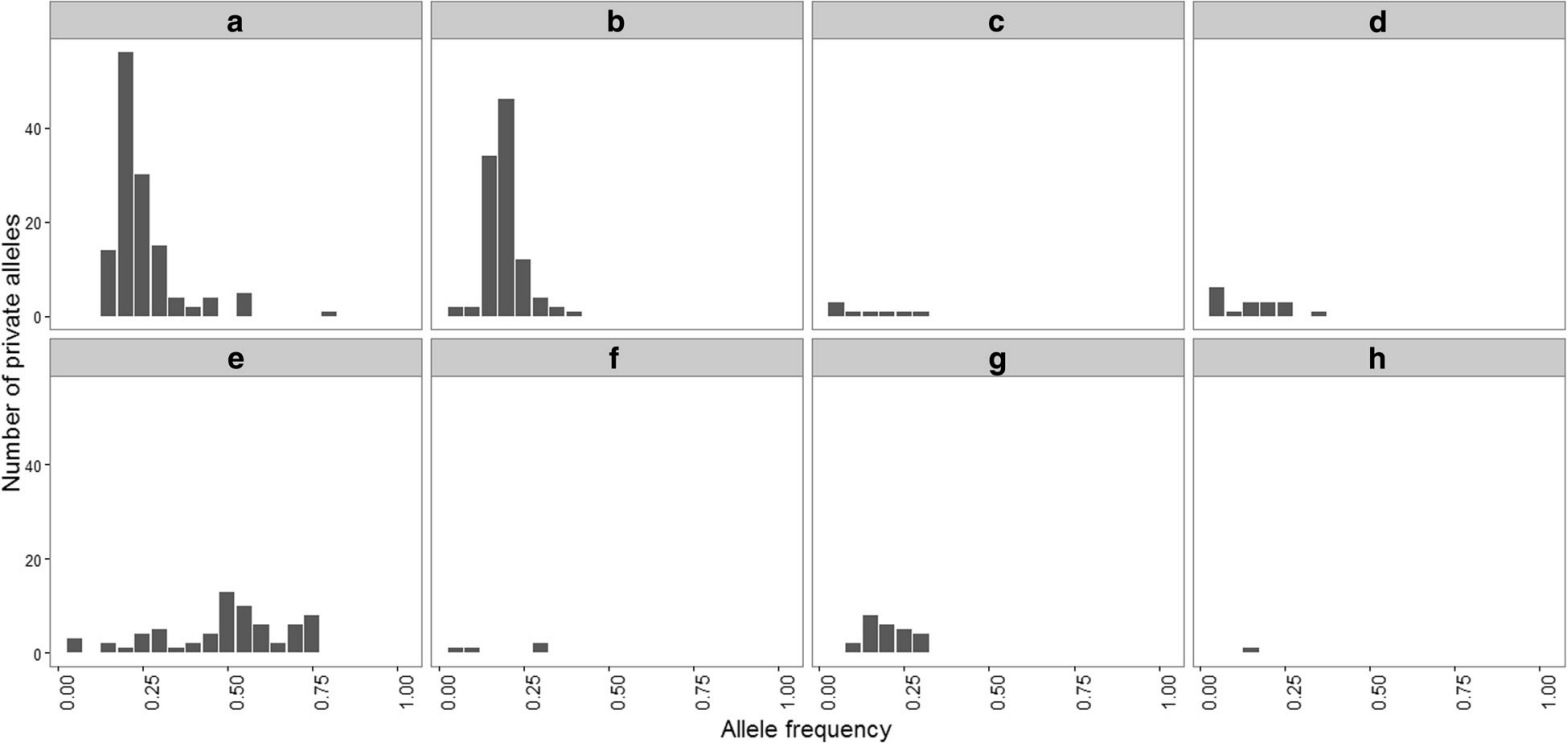
The distribution of private allele frequencies per putative marine population. **a** CA01, **b** CA02, **c** CA03, **d** OR01, **e** OR02, **f** WA01, **g** BC01, **h** AK01. See Table 1 for label meanings

All marine samples exhibited SGV, ranging from an average of 0.82% (AK01) to 1.22% (OR01) of the total SNPs genotyped in a given population; however, the pool of SGV varied from California to Alaska (Table 2). Stickleback from each marine location contained multiple private alleles (alleles found only at that location) (Fig. 3) and were polymorphic for a portion of the variant loci (loci that were polymorphic in at least one marine site). Polymorphism among variant loci varied from 57% (AK01) to 80% (OR01). For variant loci, the average frequency of the major allele (present in > 50% of all sequenced stickleback) ranged from 88% (OR02) to 93% (AK01) (Additional file 1: Figure S1), suggesting that the frequencies of SGV also differed among locations. Heterozygosity ranged from 0.10 (AK01) to 0.17 (OR02) for variant loci. Population-level average F_IS_ over all variant loci varied from 0.032 (CA01) to 0.064 (OR01) (Table 2). Although F_IS_ was close to 0 for most loci, it approached 1 for a few loci (Additional file 1: Figure S2).

### Population genetic structure

All pairwise comparisons of global F_ST_ significantly exceeded 0 (*p* < 0.001), and ranged from 0.020 to 0.181 (Table 3). Pairwise F_ST_ between the northern marine groups (WA01, BC01, and AK01) were all small (< 0.05), although other comparisons showed moderate (between 0.05–0.15), and three showed great (between 0.15–0.25) differentiation.

**Table 3:** Pairwise geographic distances (in km, above the diagonal) and global pairwise Weir and Cockerham F_ST_ (below the diagonal). All pairwise F_ST_ are significantly greater than 0

	CA01	CA02	CA03	OR01	OR02	WA01	BC01	AK01
CA01		241.68	583.77	884.70	1137.12	1615.60	1532.21	4036.73
CA02	0.096		342.09	643.02	895.44	1373.92	1290.53	3795.05
CA03	0.110	0.059		300.93	553.35	1031.83	948.44	3452.96
OR01	0.121	0.063	0.044		252.42	730.90	647.51	3152.03
OR02	0.181	0.134	0.100	0.082		478.48	395.09	2899.61
WA01	0.146	0.082	0.079	0.045	0.142		206.75	2711.27
BC01	0.157	0.095	0.094	0.056	0.159	0.027		2504.52
AK01	0.096	0.052	0.058	0.032	0.092	0.020	0.022	

**Fig. 4:**
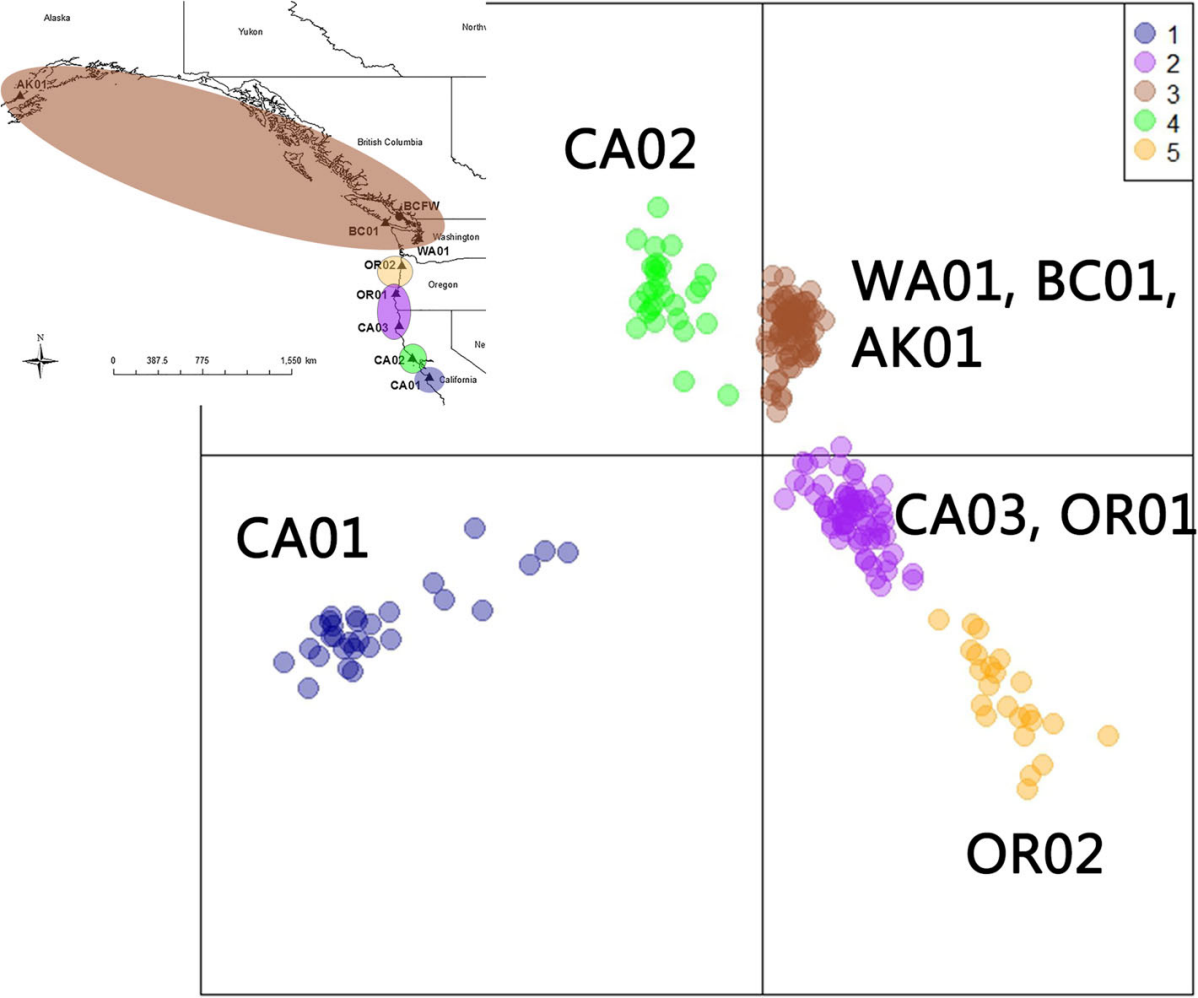
*Adegenet*-identified clusters for *k* = 5. Inset shows hypothetical range of each cluster. Note that the cluster identified as CA03, OR01 contains one AK01 and seven OR02 individuals. See Table 1 for label meanings

Significant population genetic structure was detected. The best supported number of clusters from the eight marine locations sampled was five (BIC = 1379, Fig. 4, Additional file 1: Table S4). The five clusters were, from south to north, CA01, CA02, CA03-OR01, OR02, and WA01-BC01-AK01. The CA03-OR01 cluster also contained seven individuals from OR02 and a single individual from AK01; otherwise individuals clustered with others from their sampling locality. The possibility of a single genetic cluster was as well-supported as ten genetic clusters (BIC = 1392). Three to six clusters had BIC values that differed little from the best-supported model. Altering the number of putative clusters revealed different population structures (Additional file 1: Table S4), with WA01 and AK01 continuing to cluster until *k* = 13. Cryptic population structure was not evidenced for any sampling locality (Additional file 1: Table S5). Even when WA01, BC01, and AK01 were included in a single analysis, *k* = 1 was the best supported cluster (BIC = 436.9). However, *k* = 2 (BIC = 438.2) and *k* = 3 (BIC = 440.2) still separated individuals by locality.

The genetic variance partitioned between clusters by AMOVA was low (19%) compared to within clusters (81%). When clusters were not considered, 33% of variation occurred between marine sites. A Mantel test of pairwise geographic distances and pairwise Weir and Cockerham F_ST_ was non-significant when all sites were included (*r* = − 0.2, *p* = 0.8). This was largely driven by the extreme distance between the WA01, BC01, and AK01 sites. If AK01 was excluded from the analysis the association between geographic and genetic distance was weakly significant (*r* = 0.5, *p* = 0.02) (Additional file 1: Figure S3).

A total of 4299 variant loci were sequenced in the “marine clusters” data set, with 2441 (CA01) to 3869 (CA03-OR01) SNPs per cluster. The CA01 sample included the most private alleles (*n* = 80), but the cluster of WA01-BC01-AK01, which individually had few private alleles (1 to 25), now had 47 (Additional file 1: Figure S4, Additional file 1: Table S6 and S7). The proportion of variant loci that were polymorphic within a cluster varied from 57% (CA01) to 90% (CA03-OR01). Observed heterozygosity for variant loci varied from 13% (CA01) to 17% (OR02). F_IS_ was lowest in southern California and highest in CA03-OR01 (Additional file 1: Table S7).

**Fig. 5:**
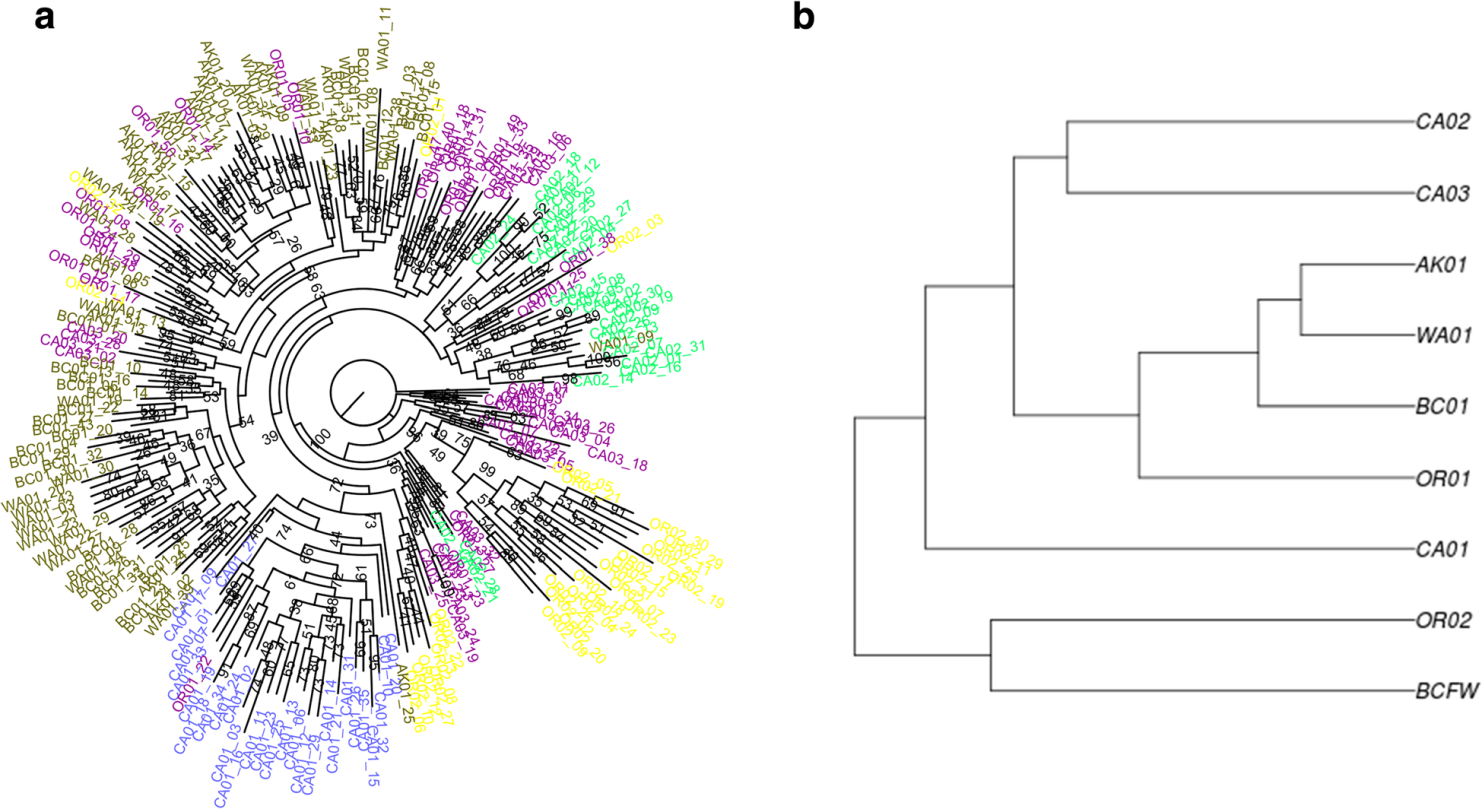
**a** Result of the phylogenetic analysis using SNPhylo. Individuals are colour-coded according to their five *Adegenet*-recognized clusters (Fig. 4). **b** Hierarchical clustering tree using *BayPass*. See Table 1 for label meanings

The phylogenetic network largely agreed with *Adegenet* assignment (Fig. 5). The network revealed greater intermixing of groups than did *Adegenet*, but WA01, BC01, and AK01 still largely clustered together and comprised a separate lineage from most southern stickleback. CA01 and CA02 constituted distinct lineages. Most OR02 individuals appeared to be derived from the CA03-OR01 clade, with 75% bootstrapping confidence. Similarly, the hierarchical clustering method grouped WA01-BC01-AK01 together, but placed OR02 as basal to all groups (Fig. 5).

### Platedness

Fish sampled from each site differed in the frequencies of plate morphs (Additional file 1: Table S2). FPK morphs comprised 100% of samples from BC01 and OR01. Four LPNK individuals were sampled from AK01, with the rest being FPK. All other sites were at least trimorphic for LPNK, PPK, and FPK. California in particular had high frequencies of LPNK stickleback, comprising 77% of samples. Five individuals from OR02 and CA01 exhibited the rare LPK morph, and a single individual from OR02 was a PPNK morph.

**Fig. 6:**
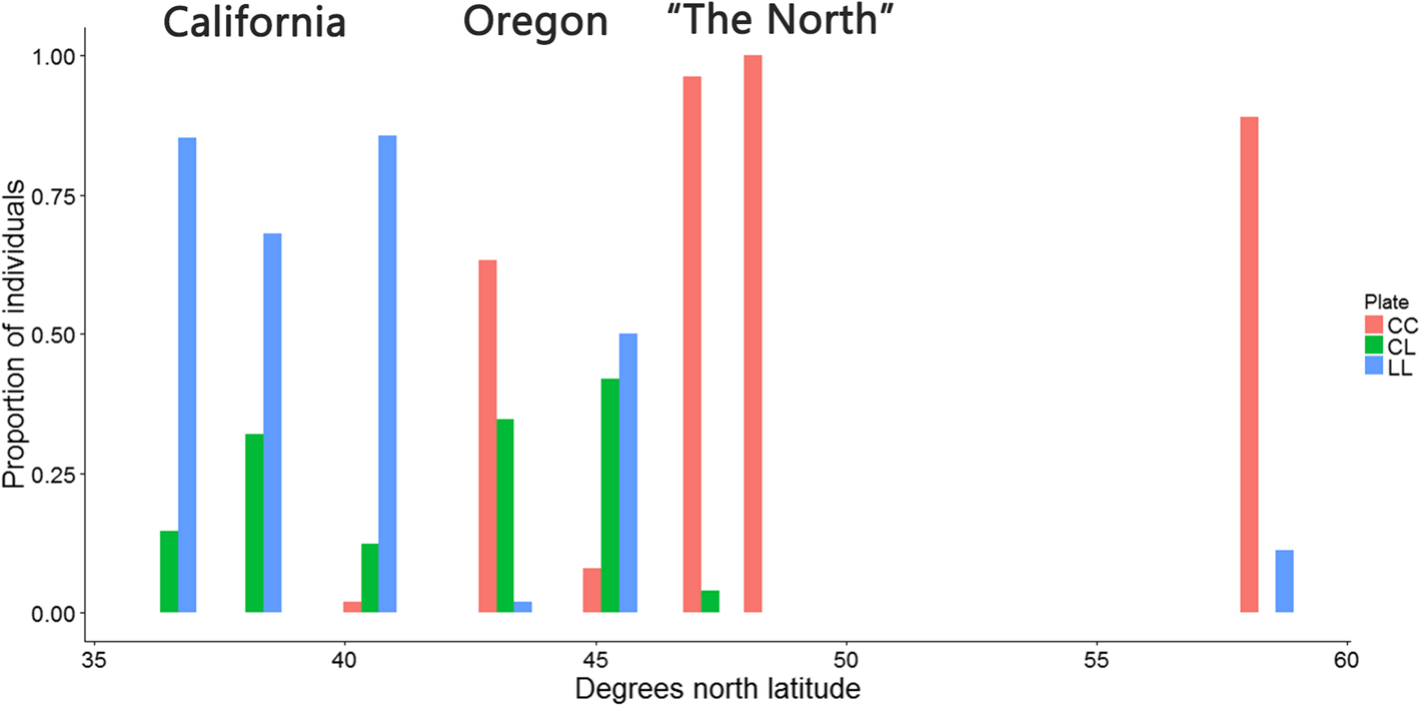
The frequency of different *Eda* genotypes using the *Stn382* marker, for each sampling site. “The North” refers to samples from Washington, British Columbia, and Alaska. CC = homozygous for the fully-plated allele. LL = homozygous for the low-plated allele. CL = heterozygous

Juvenile and adult plate morphs could be estimated using *Stn382* genotypes (Fig. 6, Additional file 1: Table S2). Only 2 of 50 WA01 individuals were heterozygous CL; the remainder were CC. Among juvenile OR01 there was a single LL, 11 CL, and 17 CC individuals. Furthermore, although all OR01 adults were FPK, six of these were CL heterozygotes. All polymorphic populations were in HWE for *Stn382*, except for AK01 (observed 24 CC, 0 CL, 3 LL, expected 21.3 CC, 5.3 CL, 0.3 LL; Chi-Squared test: 1 d.f., *p* = 0) (Additional file 1: Table S2).

### Morphometrics

**Fig. 7:**
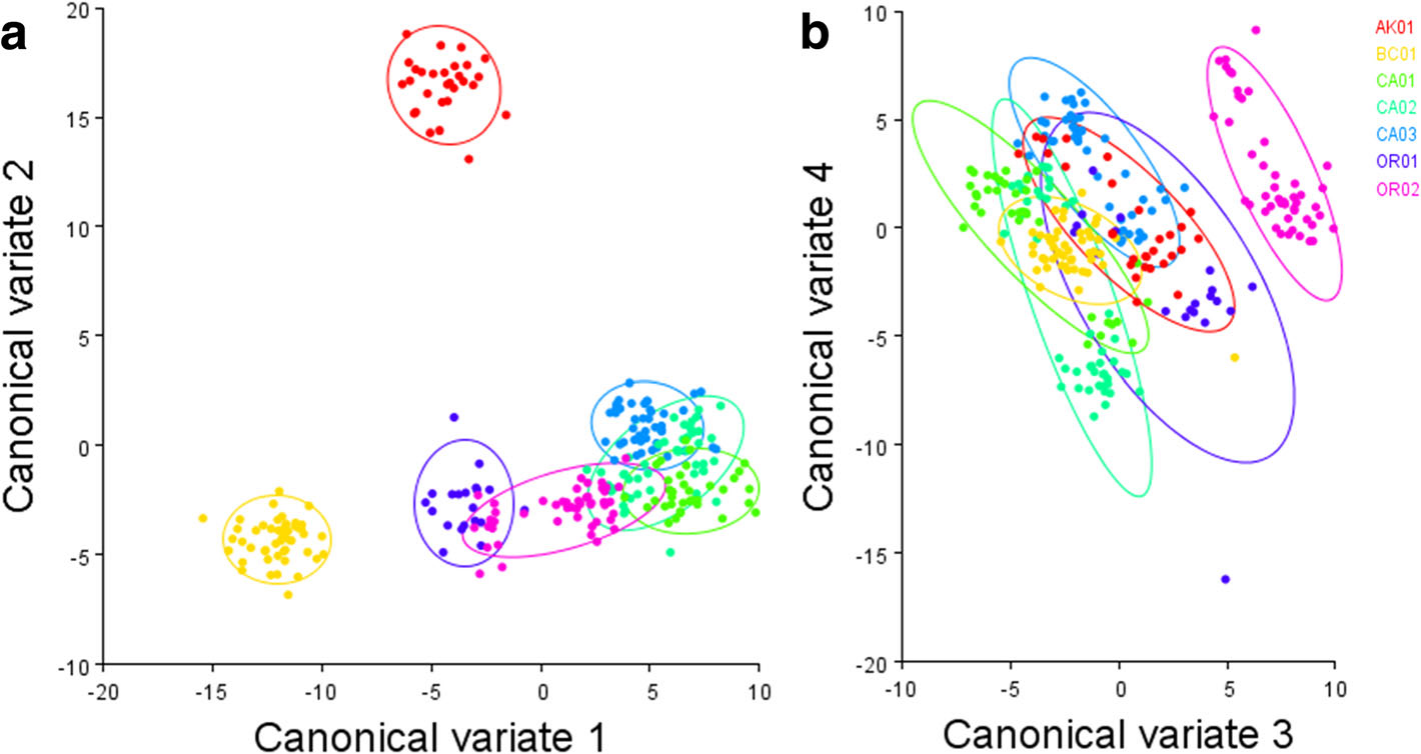
**a** Canonical Variate (CV) 1 vs CV2, and **b** CV3 vs CV4 for body shape. See Table 1 for label meanings

**Fig. 8:**
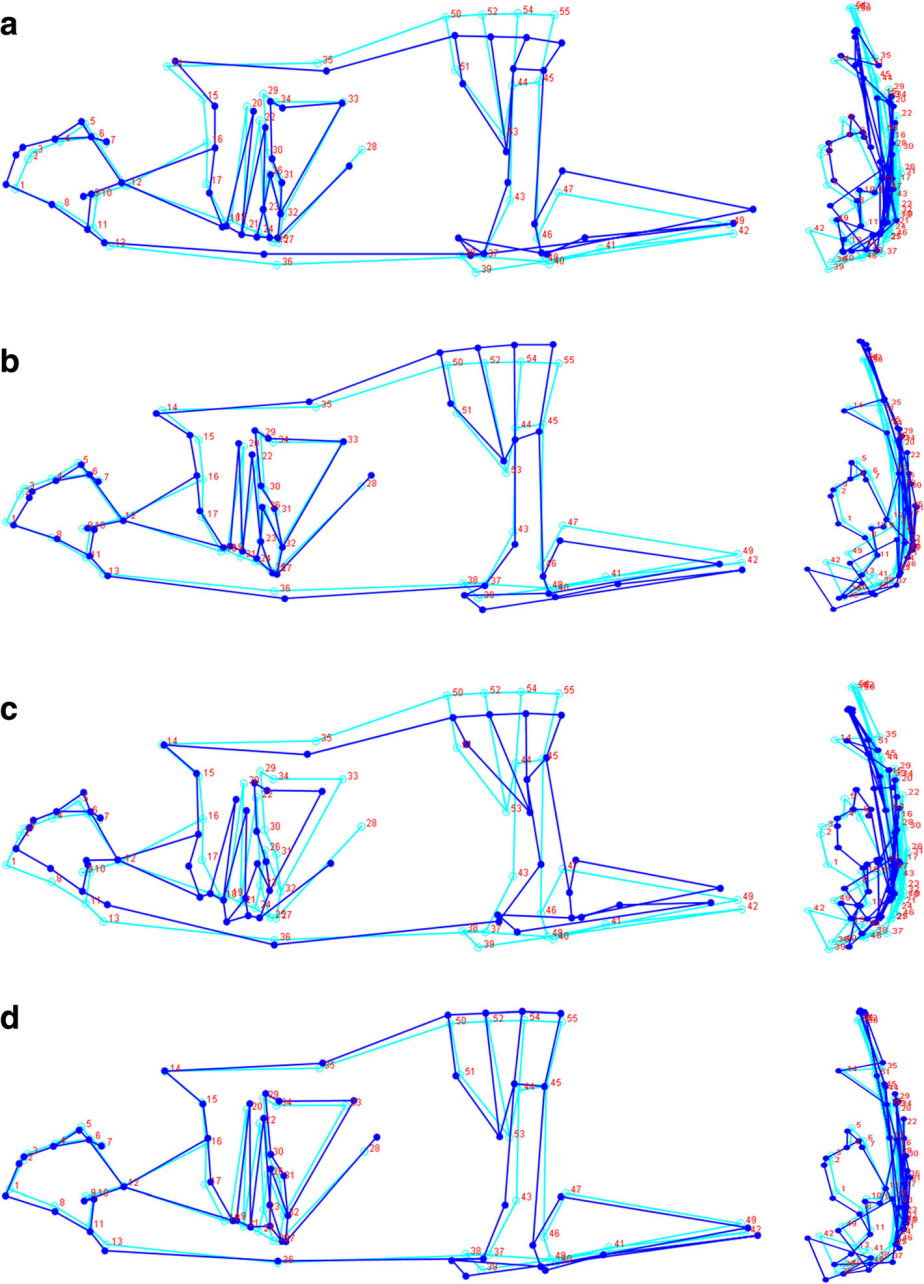
Wireframes of stickleback oriented (left) left laterally, showing the head and anterior tip of the pelvic spine situated left to right, and (right) superiorly. **a** CV1 for a BC01-type body shape; **b** CV1 for a CA01-type body shape; **c** CV2 for an AK01-type body shape; **d** CV2 for a BC01-type body shape. Light blue wireframe shows the consensus morphology, while dark blue shows the conformational change

Phenotypes varied extensively among sites. The first eight Principal Components explained 71% of all phenotypic variance (Additional file 1: Figure S5). The first two Canonical Variates (CVs) explained 57% of the variation among combinations of site and sex, and the first four CVs explained 77% (Additional file 1: Table S8). CV1, after accounting for differences in centroid size, revealed that BC01 fish had narrow, streamlined bodies with dorsal and ventral landmarks both shifting inward relative to the consensus fish (Figs. 7 and 8). Californian fish were grouped close together on CV1 and had squatter, less streamlined bodies with dorsal and ventral landmarks shifted away from one another relative to the consensus. AK01, OR01 and OR02 had intermediate phenotypes between BC01 and California. CV2 showed a gradual transition from CA01 to BC01, but AK01 was clearly distinct from all other sites along this axis. AK01 showed substantial dorsolateral and anterior-posterior constriction of the body relative to all other sites (Figs. 7 and 8).

The sexes differed morphologically at all sites except AK01 (Additional file 1: Table S9), but the sexes still largely grouped together according to sampling location. CA02 and OR02 were exceptions, with males from both sites clustering with OR01 males. Similarly, CA01 and CA02 females had morphologies that were not significantly distinct.

The DFA revealed that most fish could be classified according to marine site (based on Procrustes distance, *p* < 0.001 for all pairwise comparisons), with a single fish misclassified (Additional file 1: Table S10). Cross-validation misclassified an average of 2.8 fish per pairwise comparison (*n* = 59 total misclassifications), but this varied from 0 to 7 (CA02 – OR02), 8 (CA03 – OR02), 9 (CA01 – CA02), and 10 (CA02 – CA03). Only three fish were misclassified when comparing sites from within an *Adegenet*-recognized cluster. Thus, most misclassifications occurred among, rather than within, genetic clusters.

### P_ST_-F_ST_ and F_STQ_-F_ST_ comparisons

**Fig. 9:**
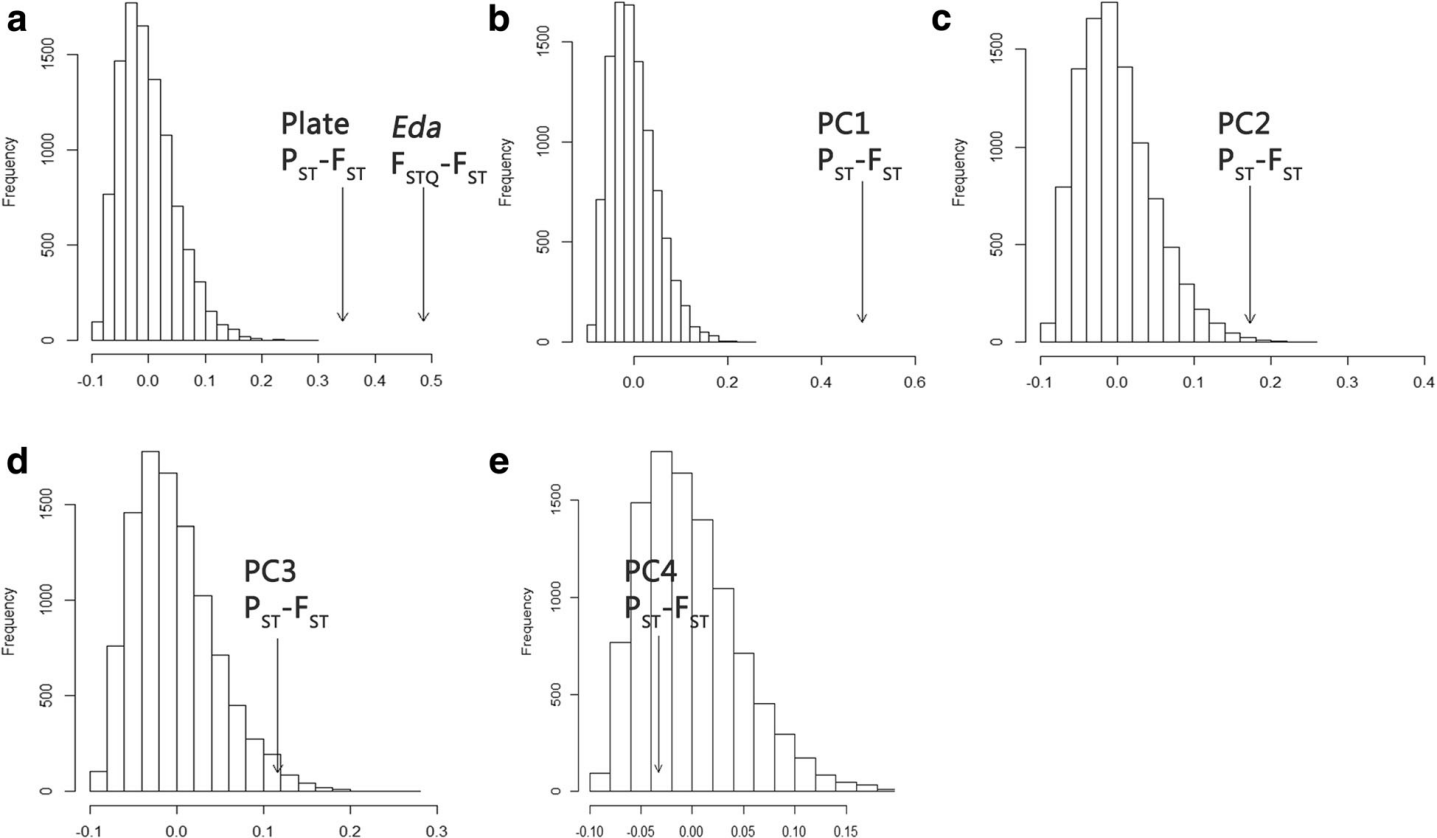
The expected neutral distribution of P_ST_ contrasted with the observed values of P_ST_-F_ST_ for **a** platedness, including F_STQ_-F_ST_ for the *Eda* allele, and principal components for body shape, including **b** PC1, **c** PC2, **d** PC3, and **e** PC4

P_ST_ was estimated as 0.46 for platedness, 0.60 for PC1, 0.29 for PC2, 0.23 for PC3, and 0.09 for PC4. F_STQ_ was 0.60. Plate P_ST_ and F_STQ_ greatly exceeded the range of the neutral P_ST_-F_ST_ distribution (*p* = 0 for both), as did P_ST_ for PC1 (*p* = 0). P_ST_ for PC2 was marginally significant but within the tail of the neutral distribution (*p* = 0.002), while P_ST_ for PC3 (*p* = 0.02) and PC4 (*p* = 0.7) were well within the neutral distributions (Fig. 9).

Of all of the Mantel tests between P_ST_, F_ST_, F_STQ_, and distance, only one was significant and two were marginally significant with corrected α = 0.05/18 = 0.0027: PC1 P_ST_ correlated positively with geographic distance, plate count P_ST_ correlated positively with F_STQ_, and, surprisingly, PC1 P_ST_ correlated positively with F_STQ_ (Table 4, Additional file 1: Figure S6 and S7). The relation between plate P_ST_ and F_STQ_ was substantiated with a generalized linear model which showed a decrease in plate number with number of L alleles (null deviance = 4813 with 277 d.f., residual deviance = 349 with 275 d.f., *p* < 0.0001, Hosmer and Lemeshow goodness of fit text: χ^2^ = − 5 × 10^− 26^, d.f. = 8, *p* = 1).

**Table 4:** The observed correlation and *p*-values (p) for Mantel tests between geographic distance, neutral genetic distance (F_ST_), phenotypic distance (P_ST_ – for plates or the first four Principal Components (PCs) of body shape), or genetic distance at *Eda* (F_STQ_)

Comparison	Observed correlation	p
Geography – neutral F_ST_	−0.29	0.7
Geography – plate number P_ST_	0.005	0.5
Geography – F_STQ_	0.26	0.1
**Geography – PC1 P**_**ST**_	**0.86**	**0.003**
Geography – PC2 P_ST_	0.003	0.21
Geography – PC3 P_ST_	−0.03	0.5
Geography – PC4 P_ST_	−0.31	0.95
Neutral F_ST_ – plate number P_ST_	0.14	0.3
Neutral F_ST_ – F_STQ_	0.14	0.2
Neutral F_ST_ – PC1 P_ST_	−0.17	0.7
Neutral F_ST_ – PC2 P_ST_	0.02	0.4
Neutral F_ST_ – PC3 P_ST_	−0.3	0.9
Neutral F_ST_ – PC4 P_ST_	−0.04	0.5
**F**_**STQ**_ **– Plate count P**_**ST**_	**0.9**	**0.002**
**F**_**STQ**_ **– PC1 P**_**ST**_	**0.5**	**0.025**
F_STQ_ – PC2 P_ST_	0.3	0.09
F_STQ_ – PC3 P_ST_	0.02	0.4
F_STQ_ – PC4 P_ST_	−0.01	0.5

**Bold** indicates significant correlations

### Selection on genetic variation in the ocean

102 of 6655 loci from the “marine sites” data set were flagged as outliers, using an XtX threshold of 15.52 (Additional file 1: Figure S8). Although variant loci were sequenced across all 21 chromosomes and an additional 65 scaffolds, outliers were only detected on 16 chromosomes and two scaffolds. Of these, 8 of 36 (22%) variant loci on scaffold 37 were outliers, followed by 13 of 178 (7%) on linkage group (LG) XXI, 11 of 184 (6%) on LGXIX, and 18 of 498 (4%) on LGIV. Of these 102 outliers, 20 were located within 16 genes (Table 5), although none of these genes have been previously studied in stickleback.

**Table 5:** Genic loci flagged as F_ST_ outliers in the marine environment

Ensembl Gene ID (ENSGACG000000)	LG	Gene name	N SNPS	XtX
15469	II	novel	1	20.36
15747	II	nfat5b	1	16.44
15682	III	glmna	2	16.15
18231	IV	abcb7	2	17.88
19242	IV	cog5	1	16.92
19472	IV	nav3	1	25.43
20913	VII	csf1rb	1	16.54
03347	VIII	podn	1	19.78
07733	VIII	csnk1g2a	1	17.90
17193	IX	nr3c2	1	17.73
05403	XI	dus1l	2	16.96
03535	XII	OVGP1	1	17.27
08321	XIX	tnni2a.2	1	19.16
08898	XIX	CERK (1 of many)	1	15.55
02461	XXI	CNTNAP2 (1 of many)	1	20.75
02744	XXI	jph1a	2	23.48

*LG* Linkage Group. XtX provides an average if the number of SNPs is > 1. The XtX threshold, above which a SNP was considered an outlier, was 15.52

### Genetic differentiation and outlier analysis for marine-freshwater comparisons

The “marine-freshwater” data set identified 132,415 loci, of which 1912 were variant. As expected, marine-freshwater divergence was high and in all but one instance was > 0.25 (“very great” differentiation). The lowest pairwise F_ST_ values involved the Oregon sites (F_ST(BCFW, OR01)_ = 0.27, F_ST(BCFW,OR02)_ = 0.18) (Additional file 1: Table S11). The maximum difference in pairwise per-locus F_ST_ estimates for a single locus was 0.991, and the average difference was 0.19. 314 (17%) SNPs had a minimum F_ST_ estimate of little genetic differentiation (< 0.05) in at least one marine-freshwater contrast, but great genetic differentiation (> 0.25) in another.

97 of 1912 loci were identified as outliers in at least one marine-freshwater comparison, using XtX thresholds of 4.9 (CA03 – FW) to 6.0 (CA01 – FW). Seventeen outlier loci resided in 13 genes (Table 6). Only 3 of 97 loci were flagged as outliers in all eight marine-freshwater comparisons, while nearly a third (31%) were flagged in only a single comparison. Five of the genic loci were outliers in seven comparisons (*tub*, *S100P*, and three novel genes), and five were outliers in only one (including a different *S100P* locus).

**Table 6:** Pairwise XtX values and outlier locus information for each of the eight marine-freshwater comparisons. LG is linkage group, although scaffolds are also included. Position refers to the nucleotide position along the linkage group

LG	Position	CA01 (6.02)	CA02 (5.25)	CA03 (4.92)	OR01 (5.85)	OR02 (5.65)	WA01 (5.34)	BC01 (5.47)	AK01 (5.10)	Total	Gene name	Marine
1	4996549					6.16				1		
1	10025443			5.79				5.79	5.51	3		
1	10025526			5.65				5.65	5.59	3		
1	11533385								5.73	1		
1	11653098			4.98						1		YES
1	11653099			5.02						1		YES
1	20898321					9.04				1		
1	21511402								6.15	1		YES
2	7863977			5.22			5.49		5.69	3	gpc51	
2	8625488	6.34	7.66	5.89	8.34		6.29	5.89	7.45	7	novel	YES
2	9813668	6.59	5.76	7.36	7.24		8.54	7.36	6.07	7	tub	
4	7681527		6.24		7.18			7.30	7.42	4		
4	7713883		5.80	7.46	7.13		6.06	7.46	6.03	6		
4	7713914		6.01	7.21	6.99		5.99	7.21	5.94	6		
4	7713937				5.93	7.24				2		
4	7728366		6.25		6.87		6.22	7.53	7.71	5		
4	7728396		6.03		6.83		5.98	7.60	7.57	5		
4	8322977				5.59					1		
4	8322992				5.69					1		
4	8323014								5.68	1		YES
4	8585549				7.19		6.44	7.36	7.47	4		YES
4	11606261						6.16			1		
4	12214334						6.59	7.41	5.84	3		
4	14370896		5.78		6.32		6.53	5.61	6.15	5		
4	14622355						6.48		5.90	2		
4	14622379				5.51		6.51		5.74	3		
4	14622421						6.34		6.06	2		
4	15435194						6.48		7.45	2	tenm1	
4	16418859							7.46	6.00	2		
4	17168113				5.98	7.20				2		
4	20971949				6.91	6.38				2		
7	6070847		5.36	5.37			5.68			3		
7	8961600			5.23						1		
7	8961654			5.27						1		
7	10052612					5.90				1	NRXN2 (1 of many)	
7	10226709				5.91					1	S100P	
7	10226775	6.44	5.48	5.64	6.67		6.69	5.64	7.46	7	S100P	
7	12677333	6.57	7.81	5.56	6.89		8.56	5.56	7.45	7		
7	12677411		5.64	5.77	7.65	9.61	6.31	5.77	5.33	7		
7	12677426	6.59	7.70	5.53	6.74		8.43	5.53	7.80	7		
7	12677430		7.57	5.34	6.97		8.25		7.52	5		
7	12677436	6.50	7.60	5.49	6.85		8.33	5.49	7.44	7		
7	12677445	6.47	7.56	5.34	6.91		8.39		7.46	6		
7	15234950				5.90					1		
7	21461506					6.79				1		YES
7	22693167					5.81				1		
8	8728904						6.48	7.33	7.34	3	csnk1g2a	YES
9	17327750					5.93				1		
9	17335552						5.68	5.49		2		
11	8223470			5.06						1		
11	11238927					6.02				1		
12	2181823						6.28		5.58	2	OVGP1	
12	2181850		5.35				6.28	5.70	6.12	4	OVGP1	
12	9470238								5.23	1	lzic	
12	9470277		5.35	5.56				5.56		3	lzic	
12	12295357	6.67	7.58	5.66	5.90		6.82	5.66	7.60	7		
12	12295366	6.47	7.73	5.73	5.93		6.81	5.73	7.55	7		
12	13222579		7.58				6.70		7.62	3		
12	13222580		5.51		7.59	9.68			5.51	4		
12	13222581		5.55		7.85	9.82			5.53	4		
12	13901004		5.55	5.78				5.78		3		
12	13901024		5.54	5.86	5.67			5.86		4		
16	6336339		5.61							1	novel	
16	6336342	6.46	6.04	4.97	6.52	7.19	6.79		5.71	7	novel	
16	12118969			5.16						1		YES
16	12119050			4.97						1		
17	6414038			5.47		5.88	5.64	5.47		4		
18	5648550	6.44	5.87	7.45	5.86	5.70	8.56	7.45	7.42	8		
18	5690662	6.51	6.03	5.47	6.14	6.33	6.77	5.47	5.78	8		
19	14803674							7.55	6.29	2		
20	5871717			5.31	7.67		6.61		6.07	4	novel	
20	5995635	6.46	7.69	7.49	7.42		6.71	7.49	5.88	7		
20	5995636	6.51	7.71	7.45	7.47		7.06	7.45	5.97	7		
20	7174610	6.58	5.93	7.39	7.36	5.95	6.13	7.39	5.91	8		
20	7634239						6.40			1		
20	7634241						6.15	5.56	5.68	3		
20	9060756			5.48	6.79	8.65	5.39	5.48		5		
20	10061253				5.56		6.73	5.67	5.75	4		
20	10061286				5.77		6.65	5.54	7.49	4		YES
20	11455928								5.32	1		
20	11455931								5.40	1		
20	11456012								5.50	1		
21	2815735		5.45	5.74				5.74	5.38	4		
21	2815747					6.21				1		
21	2957590			4.94						1	RAMP3	
21	6689655						6.13		5.90	2		
21	6689742						6.01		5.63	2		
27	3491221		5.57	5.85				5.85	5.51	4		
27	3524707	6.49	7.60	7.43	7.34		8.42	7.43	7.44	7	novel	
27	4397445	6.32	5.66	7.39	7.12		6.55	7.39	7.52	7		
27	4468817		7.64	7.24	6.82		8.43	7.24	7.51	6		
37	23177				6.08		6.05		5.72	3		YES
37	253614				5.83		6.44	5.68	5.82	4		YES
47	326567			5.38			5.43			2		
47	326633			5.28			5.44			2		
47	579296		5.63	7.56	5.79		6.56	7.56	5.69	6		
47	579344		5.77	7.41	5.84		6.68	7.41	5.76	6		

CA01 CA02 etc. identifies the marine group that was compared to the Brannen Lake British Columbia freshwater population. The number in brackets () after each population identifier is the XtX threshold value used for that marine-freshwater comparison above which a locus was flagged as an outlier. If the locus was an outlier in that comparison its XtX value is given; otherwise the cell is blank. Total refers to the number of comparisons for which that locus was flagged as an outlier. If Marine = YES that same locus was also flagged as an outlier in the marine environment

Surprisingly, 12 of the loci flagged as outliers in the “marine sites” data set were also flagged as outliers in at least one marine-freshwater comparison (Table 6), including a novel gene on LGII (*n* = 7 comparisons), and *csnk1g2a* (*n* = 3 comparisons). The gene *OVGP1* was also flagged as containing outlier loci in both analyses, but different loci were flagged in each case.

## Discussion

### Marine stickleback exhibit between-population genetic variation

The significance of SGV for parallel evolution depends on its occurrence in the ancestral populations. Marine stickleback harbour SGV, and it differs between populations. Differences in gene expression between two marine BC populations suggested this possibility [10], but here it has been quantified across an extensive latitudinal range. The extent of SGV, 0.8–1.2% of all sequenced loci, is intermediate to that reported from other studies [34, 70]. Nucleotide diversity varied from 0.0016 to 0.0027, consistent with results from Alaska (0.0022 and 0.0025 [20]) and slightly lower than that reported from Oregon (0.003–0.0036 [34]). All marine locations harboured some degree of private alleles, even after ignoring minor alleles at < 2% frequency, suggesting that not just frequencies of SGV but also content of SGV can differ from site to site. Furthermore, the best-known example of SGV, *Eda*, was present at varying frequencies between populations and is likely under selection in the marine environment. Such variation in the content and frequency of SGV, in turn, led to compelling evidence for population genetic structure. Marine threespine stickleback showed substantial population genetic structure along the Pacific coast of North America. Although F_ST_ values (average F_ST_ = 0.088) were generally lower than those reported for marine-freshwater divergence (e.g. [20, 34, 71]), they were higher than those reported for other marine stickleback populations along the North American Pacific coast (two Alaskan populations: F_ST_ = 0.0076 [20]; three Oregonian populations: F_ST_ = 0.007 [34]). However, they align with studies from Europe [39, 40, 72].

Five genetic clusters were identified for the eight sampled localities, although structuring was hierarchical, with some clusters more genetically diverged than others. The most widespread cluster occupied > 2700 km of coastline from Washington to Alaska. Marine stickleback from this genetic cluster have been well characterized, with genetic divergence reported to be low between populations separated by up to 1000 km [20, 33, 73]. Such low structuring between proximate marine populations has led to a basic assumption in stickleback literature that marine stickleback exhibit little genetic or phenotypic diversity globally (e.g. [27–29, 64, 74]) – an assumption supported by the iconic image of distinct freshwater stickleback forms radiating from a single fully-plated marine stickleback type (e.g. [75]). In contrast, results from a broad range suggest that such generalizations should be restricted to the northern genetic cluster – and even it contains morphological and genetic differentiation that could be adaptively significant.

The southern genetic clusters were sequentially separated by a few hundred kilometres, well within the migratory ability of marine stickleback [76–78]. IBD was evident only after removing AK01 from the dataset, suggesting that limited migration could explain patterns of divergence between the southern genetic clusters. However, IBD needs to be interpreted with caution, as geographic distance was correlated with latitude, and latitudinal variation can be associated with environmental clines [79]. Whatever the causes that shape genetic variation between stickleback populations, the distribution of SGV among different marine populations affects inference about the source and pace of selection in the freshwater environment, and complicates attempts to uncover loci that are under selection in derived populations.

### Marine stickleback exhibit between-population phenotypic variation

Marine stickleback are generally considered to be fully-plated (e.g. [27]), yet the *Eda* genotype for low-platedness has an ancient marine origin [18]. The low-plated allele has been hypothesized to exist in the marine environment as SGV only when transported from the freshwater environment [17] or when masked by marine modifying alleles [18]. If the low-plated allele exists at low frequencies in the ocean, behaviours that facilitate the movement of low-plated marine stickleback into fresh water could also account for the consistent colonization of rare low-plated stickleback in lakes and streams [80]. We found substantial variation in the frequency of the low-plated allele, to the point that it was the major allele in some Californian and Oregonian populations, but was absent from BC01. This finding is consistent with other records of low-plated marine stickleback in California ([81], but see [18, 82]) and high frequencies of low-platedness in European marine stickleback [61, 74, 83–85]. However, it contrasts with other Pacific and Atlantic North American studies that focussed on northern sites [86–91].

The observed SGV at *Eda* could affect the rate at which adaptation to lakes occurred in the past. Indeed, it may explain why reduced plate size has evolved in some freshwater populations, rather than reduced plate number, as an alternative strategy that may have been required in the absence of SGV at *Eda* [92, 93]. Such variation at *Eda* is a particularly striking reminder that the function of full-platedness in marine stickleback remains unknown (for an example of its possible use, demonstrated in freshwater populations, see [94]).

Marine stickleback body shape also varied extensively along the coast. Californian populations tended to have squatter body shapes that appeared to be less streamlined than their northern counterparts. The functional significance of these differences requires testing – but it is interesting that the streamlined fish were from a single genetic cluster, while the squat Californian fish exhibited significant population structuring between neighbouring localities. This potentially indicates extensive migration along the northern coast that is not mirrored in the south. Jamniczky et al. [95] reported considerable morphological divergence between neighbouring sampling sites in British Columbia – groups presumably with little to no genetic divergence, implicating plasticity as a driver of morphological variation. Morris et al. [79] similarly reported variation in vertebral number and standard length with latitude. However, two related analyses suggest that selection may also play a role in shaping phenotypic diversity.

Based on DeFaveri and Merilä’s [40] method, Pacific coast stickleback exhibited selection for platedness similar to that observed for Baltic Sea stickleback. There was also suggestive evidence for selection on PC1 of body shape, which largely corresponded to CV1 – more streamlined northern fish, more squat southern fish. The role of plasticity in affecting these results remains to be determined. PC1 was marginally associated with *Eda* genotype. To our knowledge, this is the first study to demonstrate pleiotropic or linked effects of *Eda* on body shape in the marine environment (for marine-freshwater or freshwater-only evidence for pleiotropy, see [96]; for other forms of phenotypes associated with *Eda* see [80, 97–101]), which could result in low-platedness being an indirect target of selection in some marine habitats. It is possible, for instance, that a pleiotropic relationship exists between body shape, *Eda*, and thermal tolerance – a possibility suggested by the relationship between low plate frequency and latitude in anadromous populations of Europe [83]. This is one of several possible explanations that requires formal testing.

Although *Eda* is the best-characterized example of SGV, the outlier analysis revealed other potential candidate genes for selection in the marine environment. Extensive differentiation was evidenced at some loci. Local adaptation despite gene flow has been found in other marine fish populations [35, 102–104], including European marine stickleback [72]; but the extent to which stickleback south of Washington exhibited population structure was unanticipated.

Smaller bodies and distinct body shapes tend to evolve in freshwater stickleback populations [105, 106], often with significant correlations between morphology and freshwater biotic and abiotic factors [107]. Given the morphological variation among marine stickleback, elucidating whether freshwater morphology is the result of plasticity, SGV, or de novo mutation requires informed decisions about what constitutes the marine ancestor.

### Inferring the source and pace of adaptation in freshwater stickleback

The choice of marine stickleback affected inference concerning the source and pace of adaptation in one freshwater population from Vancouver Island. Few outlier loci were consistently recovered in all marine-freshwater comparisons; had only a single marine population been used in this study, the outliers reported would differ depending on which marine population had been chosen. Many studies involve comparisons between geographically proximate marine and freshwater stickleback pairs (e.g. [21, 22]), presumably to account for the possibility of population structure in the marine environment. Yet F_ST_ was the lowest when the freshwater population was paired with a geographically distant population from northern Oregon, a finding that is difficult to reconcile with the assumption that the nearest marine population is the most suitable ancestral type. The occurrence of Japanese mtDNA haplotypes in Haida Gwaii lake populations that are not present in Haida Gwaii marine populations [108, 109] suggests that this may not be an isolated incident. Similarly, several studies have noted, but not explained, the fact that northern marine stickleback are genetically more similar to southern than northern freshwater stickleback [17, 21, 22].

Nearly one third of outlier loci were only outliers for a single marine-freshwater comparison. Thus, one’s choice of marine population could produce spurious inferences about the loci under selection, or miss true candidate genes. Clearly more information is needed going forward about the relationship between marine and freshwater stickleback.

Inferring the role of SGV during adaptive divergence in ancestral-derived comparisons requires three conditions to be met, for which we have provided varying evidence. The first, that colonists had to contain the same variants as present in the ancestral population, could not be tested here. Second, the contemporary ancestor has to have been relatively evolutionarily static. Both phenotypic and genetic evidence demonstrates that this condition does not hold for marine stickleback. Contemporary marine populations have diverged phenotypically in a manner beyond neutral evolutionary expectations, and genetically in a way partially explicable by selection. This means that contemporary marine threespine stickleback populations are genetically and phenotypically distinct from their own ancestors – and it was these ancestors that also originally colonized lakes and streams along the coast. Thus the term “contemporary ancestor” is a misnomer, as contemporary marine threespine stickleback populations do not reflect the ancestral condition. Interpretations of freshwater stickleback evolution need to be tempered by marine stickleback evolutionary history.

Third, the ancestral population has to have been properly characterized. Eastern Pacific stickleback exhibit some population genetic structure, although consistent with other reports [20, 33] stickleback north of Oregon constitute a single genetic population. This means that along much of the coast freshwater environments were likely colonized by distinct marine stickleback populations, which differed in SGV frequency and content. Furthermore, it is likely that marine stickleback have exhibited range contractions and expansions along the southern and northern coasts throughout their evolutionary history, most recently in the north after the last glacial retreat [110]. This means that there is no a priori reason to expect that a marine population currently proximate to freshwater populations are descendants of the ancestors of those freshwater populations. Population structuring and evolutionary history thus changes our understanding of the ancestral condition of marine stickleback, and requires that we carefully consider the use of contemporary marine populations when addressing evolutionary questions.

## Conclusions

Studies that compare marine and freshwater stickleback may need to adjust their methodologies in light of marine stickleback variation. The typical image in textbooks is of a single marine stickleback form from which numerous freshwater forms radiate [75]. Our data suggests that there is phenotypic and genetic variation in marine threespine stickleback which likely impacts freshwater stickleback diversification – to assume a single marine form is no longer tenable. Furthermore, assuming ancestral status for the marine population most geographically proximate to the freshwater population of interest is problematic, unless it can be directly demonstrated (e.g. [111]). So where does this leave the comparative method? One possibility would be to reconstruct the genotype of the ancestor to all eastern Pacific marine stickleback – but this would ignore the important role that local variation has played in freshwater stickleback evolution. Another possibility could be to conduct larger-scale geographic sampling than has heretofore been done, of both marine and freshwater forms, in order to determine a more thorough evolutionary history of this species. Then, having taken evolutionary relationships into account, comparisons can be made using better-justified “contemporary ancestors”.

“Contemporary ancestors” are used in a number of systems [3–11] for addressing evolutionary questions. They are particularly useful for determining the role of SGV during evolution, and for identifying the alleles involved in adaptation to new environments. Clearly, care must be exercised in characterizing these proxies of the ancestral form, as unaccounted population structure and current evolution can lead to spurious interpretations of adaptation. Whether the lessons from stickleback apply to other species with smaller geographic distributions or more limited opportunities for gene flow waits to be seen.

## Additional file

Additional file 1: Additional tables and figures. This document contains information supporting the main text, including: the list of landmarks used for 3D morphometrics, population-specific details on sex and platedness, additional information pertaining to methodology, the distributions of major allele frequencies and FIS per population, isolation-by-distance analyses, details regarding *Adegenet*-recognized clusters, CVA and DFA morphometrics results, additional Mantel tests, global pairwise FST values for each marine-freshwater comparison, and results pertaining to the outlier analysis. (DOCX 1008 kb)

## Abbreviations

AK: Alaska
AMOVA: Analysis of molecular variance
BC: British Columbia
BIC: Bayesian Information Criteria
C: Fully plated *Eda* allele
CA: California
CV: Canonical variate
CVA: Canonical variate analysis
DAPC: Discriminant Analysis of Principal Components
DFA: Discriminant Function Analysis
*Eda*: *Ectodysplasin*
F: Female
FPK: Fully plated with keel
F_STQ_: Genetic divergence at *Eda* allele
FW: Freshwater
GLM: Generalized linear model
HWE: Hardy-Weinberg equilibrium
IBD: Isolation-by-Distance
km: Kilometer
L: Low plated *Eda* allele
LG: Linkage group
LPK: Low plated with keel
LPNK: Low plated without keel
M: Male
OR: Oregon
PC: Principal Component
PCA: Principal Components Analysis
POD: Pseudo-observed data set
PPK: Partially plated with keel
PPNK: Partially plated without keel
P_ST_: Estimate of phenotypic divergence
SGV: Standing genetic variation
SL: Standard length
WA: Washington
μCT: Micro-computed tomography
μm: Micrometer
σ^2^_B_: Between-population component of phenotypic variance
σ^2^_W_: Within-population component of phenotypic variance
