# Supplementary material for: Contemporary ancestor? Adaptive divergence from standing genetic variation in Pacific marine threespine stickleback
Source: BMC Evol Biol. 2018 Jul 18;18:113. doi: 10.1186/s12862-018-1228-8 (PMC6052716; doi:10.1186/s12862-018-1228-8)

Table S1 - List of landmarks used for 3D morphometrics.

| Landmark | Description |
| --- | --- |
| 1 | Anterior tip of dentary |
| 2 | Anterior tip of premaxilla |
| 3 | Anterior tip of maxilla |
| 4 | Anterior corner of nasal ventrolateral process |
| 5 | Dorsal corner of nasal-lateral ethmoid suture |
| 6 | Dorsal maxima of lachrymal |
| 7 | Lachrymal-prefrontal suture on orbital |
| 8 | Anterior tip of articular |
| 9 | Ventral maxima of lachrymal |
| 10 | Dorsal tip of articular |
| 11 | Ventral-most tip of articular |
| 12 | Lachrymal-second orbital suture |
| 13 | Anterior tip of preoperculum |
| 14 | Dorsal-most tip of supraorbital |
| 15 | Ventral-most tip of sphenotic |
| 16 | Dorsal-most tip of third suborbital |
| 17 | Posterior minima of third suborbital |
| 18 | Ventral-most tip of third suborbital |
| 19 | Anterior minima of preoperculum |
| 20 | Anterior dorsal-most tip of preoperculum |
| 21 | Posterior maxima of preoperculum, first ridge |
| 22 | Ventral dorsal-most tip of preoperculum |
| 23 | Dorsal-most tip of interoperculum |
| 24 | Ventral maxima of preoperculum, second ridge |
| 25 | Ventral-most tip of interoperculum |
| 26 | Dorsal-most tip of suboperculum |
| 27 | Ventral maxima of suboperculum |
| 28 | Posterior tip of suboperculum |
| 29 | Dorsal-most tip of operculum |
| 30 | Anterior maxima of operculum |
| 31 | Anterior minima of operculum |
| 32 | Ventral-most tip of operculum |
| 33 | Posteriodorsal tip of operculum |
| 34 | Opercular hinge angle |
| 35 | Posterior tip of pterotic |
| 36 | Anterior tip of ectocoracoid |
| 37 | Posterior tip of ectocoracoid |
| 38 | Anterior tip of pelvic plate |
| 39 | Anterior midline of pelvic plate at suture point |
| 40 | Minima of pelvic plate at trochlear joint |
| 41 | Maxima of pelvic process |
| 42 | Posterior tip of pelvic process |
| 43 | Anterior minima of ascending process of pelvic plate |
| 44 | Anteriodorsal maxima of ascending process of pelvic plate |
| 45 | Posteriodorsal maxima of ascending process of pelvic plate |
| 46 | Posterioventral maxima of ascending process of pelvic plate at trochlear joint |
| 47 | Dorsal-most tip of pelvic spine |
| 48 | Ventral-most tip of pelvic spine |
| 59 | Posterior tip of pelvic spine |
| 50 | Midline of plate 4 at lateral pores |
| 51 | Ventral tip of plate 4 |
| 52 | Midline of plate 5 at lateral pores |
| 53 | Ventral tip of plate 5 |
| 54 | Midline of plate 6 at lateral pores |
| 55 | Midline of plate 7 at lateral pores |

Table S2 – The numbers of each sex (M = Male, F = Female), plate morph (FPK = fully-plated, PPK = partially-plated, LP = low-plated, K = keel present, NK = keel absent), and *Stn382* genotype (C = fully-plated allele, L = low-plated allele) sampled from California to Alaska. The number of fish within each category that made it through the *Stacks* pipeline are indicated in brackets. Note that not every fish could be both genotyped and phenotyped. * Indicates violation from HWE for *Stn382*.

| Population | M | F | FPK | PPK | LPNK | PPNK | LPK | CC | CL | LL |
| --- | --- | --- | --- | --- | --- | --- | --- | --- | --- | --- |
| CA01 | 26 (23) | 9 (6) | 2 (1) | 5 (4) | 25 (21) | 0 (0) | 3 (3) | 0 (0) | 5 (5) | 29 (24) |
| CA02 | 20 (8) | 30 (20) | 7 (4) | 9 (4) | 34 (20) | 0 (0) | 0 (0) | 0 (0) | 16 (8) | 34 (20) |
| CA03 | 31 (17) | 18 (11) | 2 (1) | 5 (3) | 42 (24) | 0 (0) | 0 (0) | 1 (0) | 6 (4) | 42 (24) |
| OR01 | 25 (14) | 25 (15) | 20 (18) | 0 (0) | 0 (0) | 0 (0) | 0 (0) | 31 (19) | 17 (9) | 1 (1) |
| OR02 | 13 (7) | 37 (23) | 12 (5) | 11 (9) | 24 (14) | 1 (1) | 2 (1) | 4 (1) | 21 (14) | 25 (15) |
| WA01 | 35 (18) | 16 (7) | NA | NA | NA | NA | NA | 49 (24) | 2 (1) | 0 (0) |
| BC01 | 47 (28) | 4 (3) | 48 (31) | 0 (0) | 0 (0) | 0 (0) | 0 (0) | 51 (31) | 0 (0) | 0 (0) |
| AK01* | 8 (6) | 23 (18) | 27 (23) | 0 (0) | 4 (1) | 0 (0) | 0 (0) | 24 (22) | 0 (0) | 3 (1) |

Table S3 - Number of reads dropped or retained by *process_radtags*.

| Measurement | N reads |
| --- | --- |
| Total sequenced reads | 249 038 733 |
| Total containing adapter | 1 839 266 |
| Ambiguous barcode drops | 40 205 180 |
| Low quality read drops | 129 991 |
| Ambiguous RAD-tag drops | 13 977 761 |
| Retained reads | 192 886 535 |
| % retained | 77.4% |

Table S4 - Bayesian Information Criteria (BIC) for clusters *k* = 2 to 8, for the SNP dataset using all eight marine sites. SNPs were called if the locus was sequenced in at least six marine groups and >75% of individuals. Members of a cluster are grouped in [ ], numbers are used if a few individuals from another locality were included in the cluster.

| Number of clusters | BIC value | Clusters |
| --- | --- | --- |
| 2 | 1385.45 | [OR02] [CA01, CA02, CA03, OR01, WA01, BC01, AK01] |
| 3 | 1380.30 | [OR02] [CA01] [CA02, CA03, OR01, 2 OR02, WA01, BC01, AK01] |
| 4 | 1379.82 | [OR02] [CA01] [CA03, OR01, 7 OR02, 1 AK01] [CA02, WA01, BC01, AK01] |
| 5 | 1378.96 | [OR02] [CA01] [CA03, OR01, 7 OR02, 1 AK01] [CA02] [WA01, BC01, AK01] |
| 6 | 1380.75 | [OR02] [CA01] [OR01, 7 OR02, 1 AK01] [CA02] [CA03] [WA01, BC01, AK01] |
| 7 | 1383.82 | [OR02] [CA01] [OR01, 7 OR02, 1 AK01] [CA02] [CA03] [BC01] [WA01, 1 BC01, AK01] |
| 8 | 1386.38 | [OR02] [CA01] [OR01, 7 OR02, 1 AK01] [CA02] [CA03] [BC01] [WA01, 1 BC01, AK01]  [4 CA02] |

Table S5 – Bayesian Information Criteria (BIC) values for *k* = 1 to 3, for each marine site treated on its own through the *Stacks* pipeline and *Adegenet*, to test for cryptic population structure. The number of Single Nucleotide Polymorphisms (SNPs) retained by *Stacks* is indicated. The final test included all fish from the *Adegenet*-recognized northern cluster. To maximize the potential differences between individuals, 22 PCs were retained despite being far higher than N/3. The lowest BIC values are shown in **bold**.

| Marine site | SNPs | *k* = 1 | *k* = 2 | *k* = 3 |
| --- | --- | --- | --- | --- |
| CA01 | 15413 | **211.388** | 213.388 | 215.277 |
| CA02 | 30492 | **204.388** | 205.175 | 206.2744 |
| CA03 | 19185 | **204.941** | 206.615 | 208.571 |
| OR01 | 21904 | **218.062** | 220.098 | 222.086 |
| OR02 | 22377 | **229.990** | 231.841 | 233.863 |
| WA01 | 17342 | **179.686** | 181.580 | NA |
| BC01 | 15416 | **226.996** | 229.160 | 231.285 |
| AK01 | 8702 | **153.336** | 154.833 | NA |
| WA01 & BC01 & AK01 | 7308 | **516.189** | 517.536 | 519.439 |

Table S6 – Pairwise F_ST_ values for *Adegenet*-recognized clusters.

|  | CA01 | CA02 | CA03 & OR01 | OR02 |
| --- | --- | --- | --- | --- |
| CA02 | 0.107 |  |  |  |
| CA03 & OR01 | 0.114 | 0.053 |  |  |
| OR02 | 0.215 | 0.169 | 0.105 |  |
| WA01 & BC01 & AK01 | 0.161 | 0.094 | 0.061 | 0.206 |

Table S7 - Full population genetic statistics when using the identified *Adegenet* clusters. (Top) For all variant positions. (Bottom) For all sites. Note that the CA03 & OR01 cluster includes a few individuals from OR02 and AK01 (see main text). Private = Private alleles. N = Number of individuals used. P = Average major allele frequency. Het_O_ = Observed heterozygosity. Het_E_ = Expected heterozygosity. H_O_ = Observed homozygosity. H_E_ = Expected homozygosity. π = Nucleotide diversity. F_IS_ = Inbreeding coefficient. Loci = Average number of loci that were sequenced. Variant = Number of loci that were polymorphic in at least one marine site. SNP = Number of Single Nucleotide Polymorphisms. % Poly = Proportion of variant loci that were polymorphic in the marine site of interest (top) or the proportion of sequenced loci that were polymorphic in the marine site of interest (bottom).

| Cluster | Private | N | % Poly | P | Het_O_ | H_O_ | Het_E_ | H_E_ | π | F_IS_ |
| --- | --- | --- | --- | --- | --- | --- | --- | --- | --- | --- |
| CA01 | 80 | 25.8 | 56.8 | 0.90 | 0.13 | 0.87 | 0.14 | 0.86 | 0.14 | 0.03 |
| CA02 | 35 | 25.3 | 68.6 | 0.90 | 0.14 | 0.86 | 0.15 | 0.85 | 0.15 | 0.03 |
| CA03 & OR01 | 14 | 54.9 | 90.0 | 0.89 | 0.14 | 0.86 | 0.16 | 0.84 | 0.16 | 0.09 |
| OR02 | 10 | 21.9 | 77.6 | 0.88 | 0.17 | 0.83 | 0.18 | 0.82 | 0.18 | 0.04 |
| WA01 & BC01 & AK01 | 47 | 66.7 | 79.3 | 0.92 | 0.12 | 0.88 | 0.13 | 0.87 | 0.13 | 0.07 |

| Cluster | Loci | Variant loci | Poly loci | % Poly | N | P | Het_O_ | H_O_ | Het_E_ | H_E_ | π | F_IS_ |
| --- | --- | --- | --- | --- | --- | --- | --- | --- | --- | --- | --- | --- |
| CA01 | 286058 | 4299 | 2441 | 0.85 | 26.3 | 0.999 | 0.002 | 0.998 | 0.002 | 0.998 | 0.002 | 0.0004 |
| CA02 | 286058 | 4299 | 2947 | 1.03 | 25.72 | 0.999 | 0.002 | 0.998 | 0.002 | 0.999 | 0.002 | 0.0005 |
| CA03/ORO1 | 286058 | 4299 | 3869 | 1.35 | 56.0 | 0.998 | 0.002 | 0.998 | 0.002 | 0.998 | 0.002 | 0.0013 |
| OR02 | 286058 | 4299 | 3335 | 1.17 | 22.1 | 0.998 | 0.003 | 0.997 | 0.003 | 0.997 | 0.003 | 0.0006 |
| WA01, BC01, AK01 | 286058 | 4299 | 3410 | 1.19 | 67.7 | 0.999 | 0.002 | 0.998 | 0.002 | 0.998 | 0.002 | 0.0011 |

Table S8 – 3D morphometric variation from Canonical Variate Analysis (CVA) among seven marine groups, scaled by the inverse of the within-group variation, for CVs 1 through 14.

| CV | Eigenvalues | % Variance | Cumulative % |
| --- | --- | --- | --- |
| 1 | 48.34 | 32.04 | 32.04 |
| 2 | 37.48 | 24.85 | 56.89 |
| 3 | 17.30 | 11.47 | 68.36 |
| 4 | 12.90 | 8.55 | 76.91 |
| 5 | 8.74 | 5.79 | 82.70 |
| 6 | 5.64 | 3.74 | 86.44 |
| 7 | 5.28 | 3.50 | 89.94 |
| 8 | 4.29 | 2.84 | 92.78 |
| 9 | 3.19 | 2.11 | 94.89 |
| 10 | 2.15 | 1.43 | 96.32 |
| 11 | 1.69 | 1.12 | 97.44 |
| 12 | 1.63 | 1.08 | 98.52 |
| 13 | 1.34 | 0.89 | 99.41 |
| 14 | 0.89 | 0.59 | 100 |

Table S9 – Procrustes distance (above diagonal) and p-values (below diagonal) for Canonical Variate Analysis (CVA) with site and sex as categorical variables. Note that BC01 had a single female and OR01 had a single individual not assigned to sex; the results of those two individuals are not shown. F = female, M = male, * = significance below 0.0005.

|  | CA01, F | CA01, M | CA02, F | CA02, M | CA03, F | CA03, M | OR01, F | OR01, M | OR02, F | OR02, M | BC01, M | AK01, F | AK01, M |
| --- | --- | --- | --- | --- | --- | --- | --- | --- | --- | --- | --- | --- | --- |
| CA01, F |  | 0.039 | 0.030 | 0.043 | 0.041 | 0.043 | 0.051 | 0.046 | 0.043 | 0.054 | 0.073 | 0.111 | 0.098 |
| CA01, M | * |  | 0.052 | 0.025 | 0.045 | 0.033 | 0.071 | 0.045 | 0.050 | 0.039 | 0.062 | 0.108 | 0.088 |
| CA02, F | 0.007 | * |  | 0.047 | 0.034 | 0.043 | 0.035 | 0.052 | 0.043 | 0.063 | 0.075 | 0.103 | 0.095 |
| CA02, M | * | * | * |  | 0.039 | 0.025 | 0.060 | 0.036 | 0.040 | 0.033 | 0.052 | 0.103 | 0.084 |
| CA03, F | * | * | * | * |  | 0.028 | 0.047 | 0.051 | 0.038 | 0.051 | 0.066 | 0.089 | 0.076 |
| CA03, M | * | * | * | * | * |  | 0.055 | 0.038 | 0.041 | 0.036 | 0.061 | 0.100 | 0.079 |
| OR01, F | * | * | * | * | * | * |  | 0.052 | 0.042 | 0.067 | 0.067 | 0.096 | 0.091 |
| OR01, M | * | * | * | 0.003 | * | * | * |  | 0.039 | 0.034 | 0.052 | 0.111 | 0.090 |
| OR02, F | * | * | * | * | * | * | * | * |  | 0.039 | 0.057 | 0.096 | 0.085 |
| OR02, M | * | * | * | * | * | * | * | 0.016 | * |  | 0.055 | 0.109 | 0.085 |
| BC01, M | * | * | * | * | * | * | * | * | * | * |  | 0.089 | 0.072 |
| AK01, F | * | * | * | * | * | * | * | * | * | * | * |  | 0.003 |
| AK01, M | * | * | * | * | * | * | * | * | * | * | * | 0.042 |  |

Table S10 – The number of individuals assigned to the wrong location based on phenotype for the Discriminant Function Analysis (DFA) (above diagonal) and cross-validation (below diagonal).

|  | CA01 | CA02 | CA03 | OR01 | OR02 | BC01 | AK01 |
| --- | --- | --- | --- | --- | --- | --- | --- |
| CA01 |  | 0 | 0 | 0 | 0 | 0 | 0 |
| CA02 | 9 |  | 1 | 0 | 0 | 0 | 0 |
| CA03 | 4 | 10 |  | 0 | 0 | 0 | 0 |
| OR01 | 0 | 3 | 2 |  | 0 | 0 | 0 |
| OR02 | 1 | 7 | 8 | 2 |  | 0 | 0 |
| BC01 | 1 | 0 | 2 | 1 | 5 |  | 0 |
| AK01 | 2 | 0 | 0 | 0 | 1 | 1 |  |

Table S11 – Pairwise F_ST_ values for each of the eight marine-freshwater comparisons. Marine-BCFW identifies the marine group that was compared to the Brannen Lake, BC, freshwater population.

| BCFW paired with | F_ST_ |
| --- | --- |
| CA01 | 0.365 |
| CA02 | 0.329 |
| CA03 | 0.308 |
| OR01 | 0.274 |
| OR02 | 0.184 |
| WA01 | 0.351 |
| BC01 | 0.373 |
| AK01 | 0.385 |

Figure S1 - The distribution of major allele frequencies for each population. (a) CA01, (b) CA02, (c) CA03, (d) OR01, (e) OR02, (f) WA01, (g) BC01, (h) AK01.


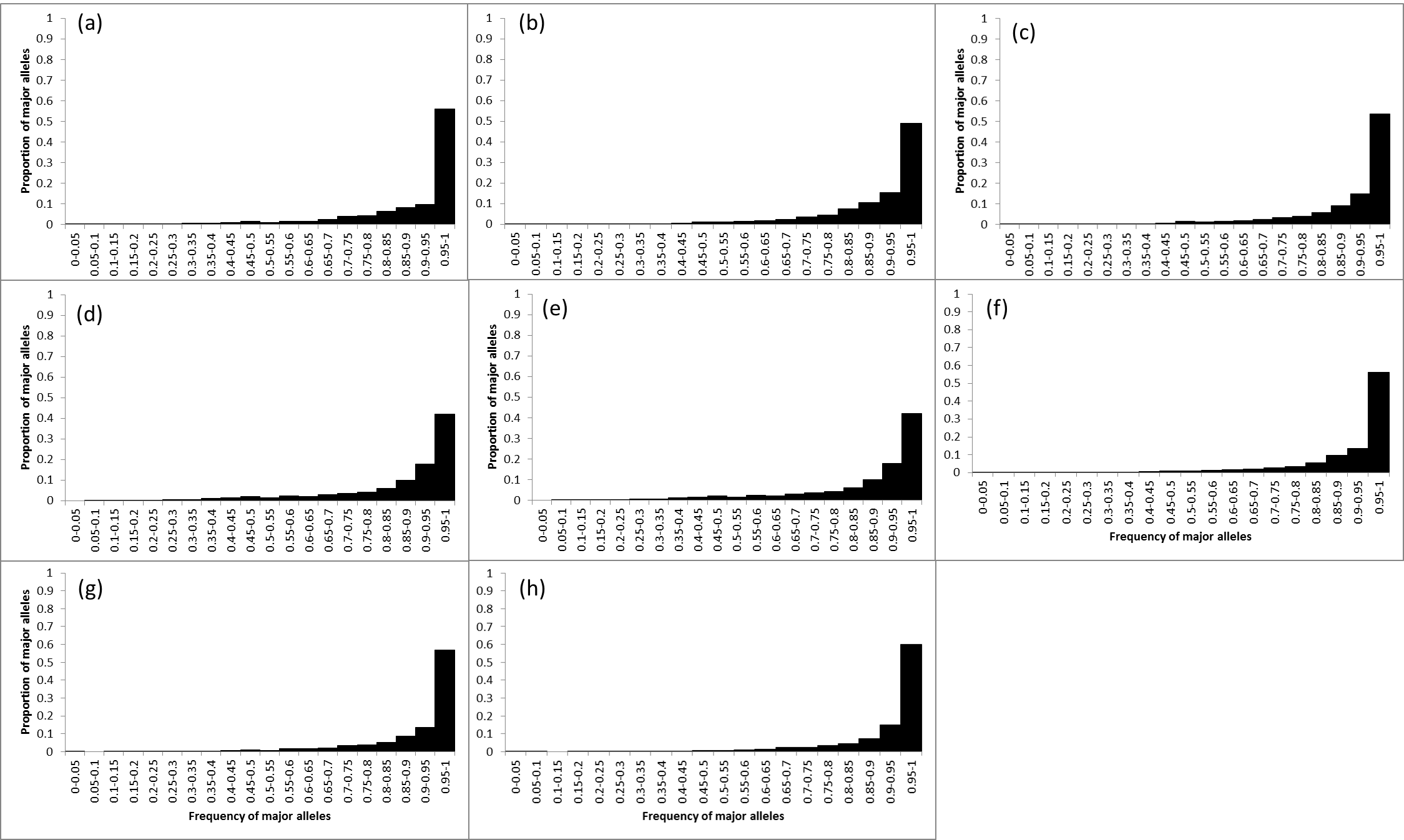


Figure S2 - The distribution of per-locus F_IS_ values for each population. (a) CA01, (b) CA02, (c) CA03, (d) OR01, (e) OR02, (f) WA01, (g) BC01, (h) AK01. Note that F_IS_ values less than -0.3 were filtered from the dataset.


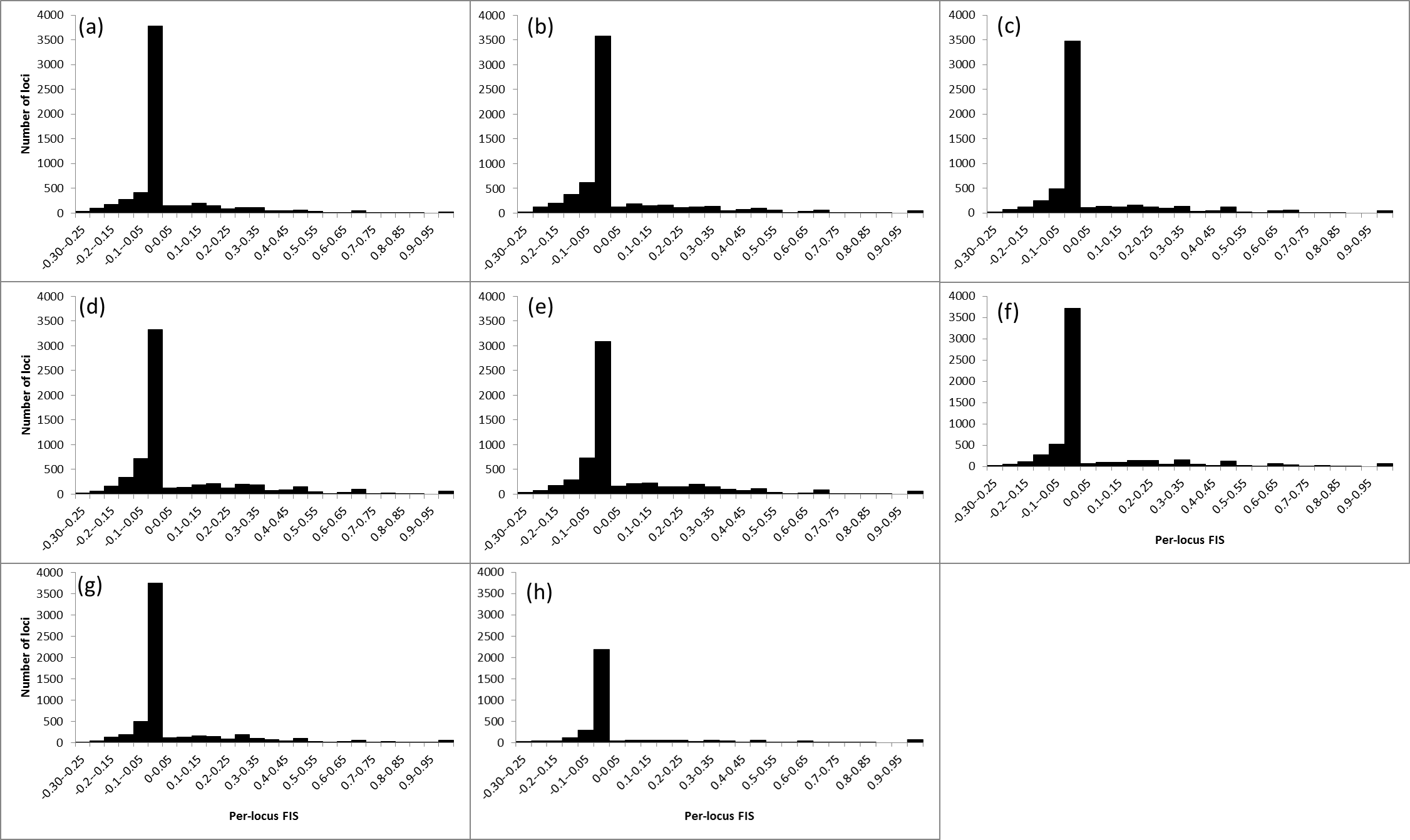


Figure S3 - Isolation-by-Distance as tested using a Mantel test of pairwise geographic distances (km) on pairwise genetic distances (Weir and Cockerham F_ST_), at 999 replications. (a) For all populations, p = 0.8. (b) Excluding AK01, p = 0.02.


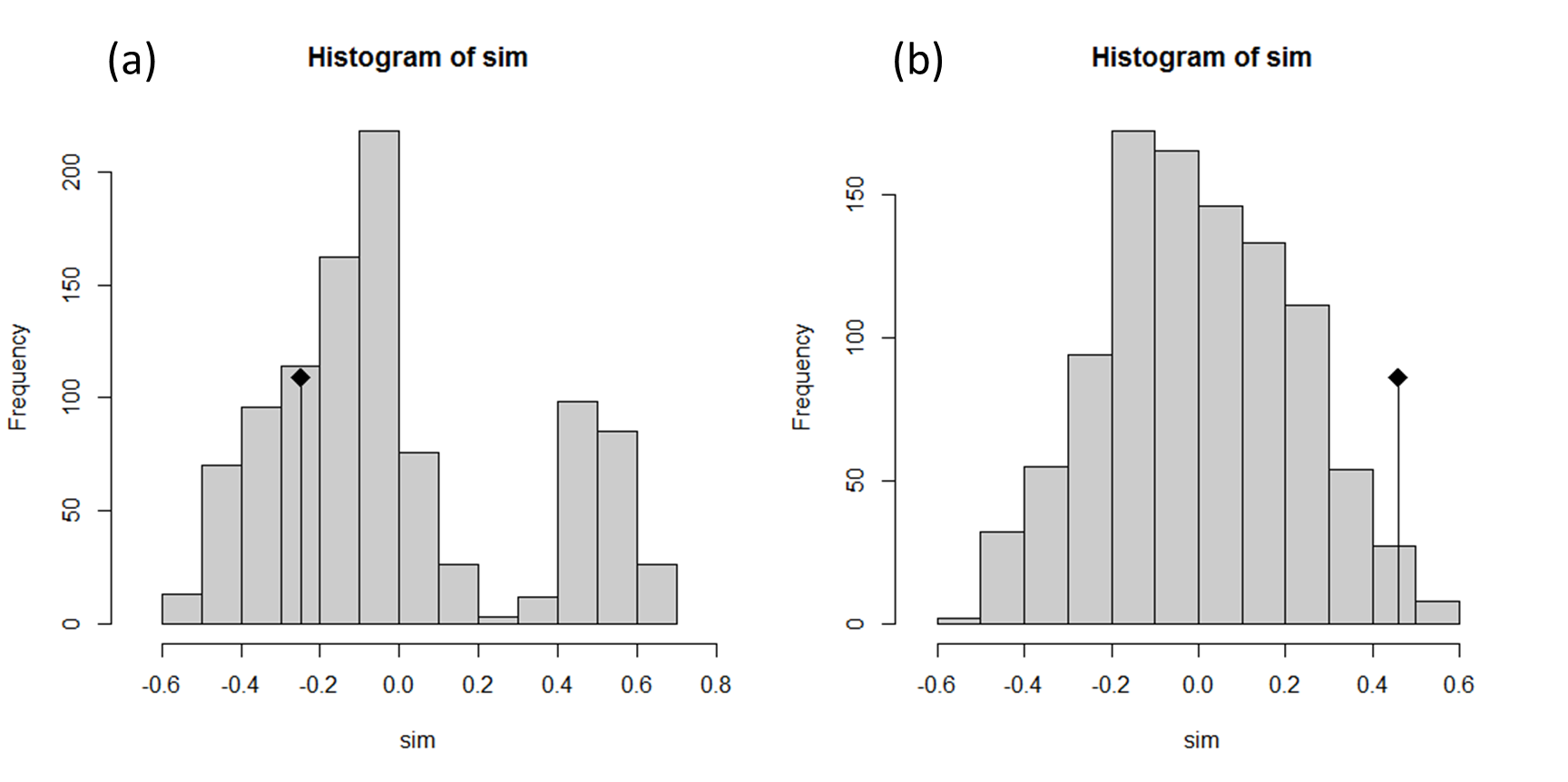


Figure S4 - The distribution of private allele frequencies when assigning individuals based on Adegent clustering at *k* = 5. (a) CA01, (b) CA02, (c) CA03 & OR01 & 7 OR02 & 1 AK01, (d) OR02, (e) WA01 & BC01 & AK01. Compare to Figure S1.


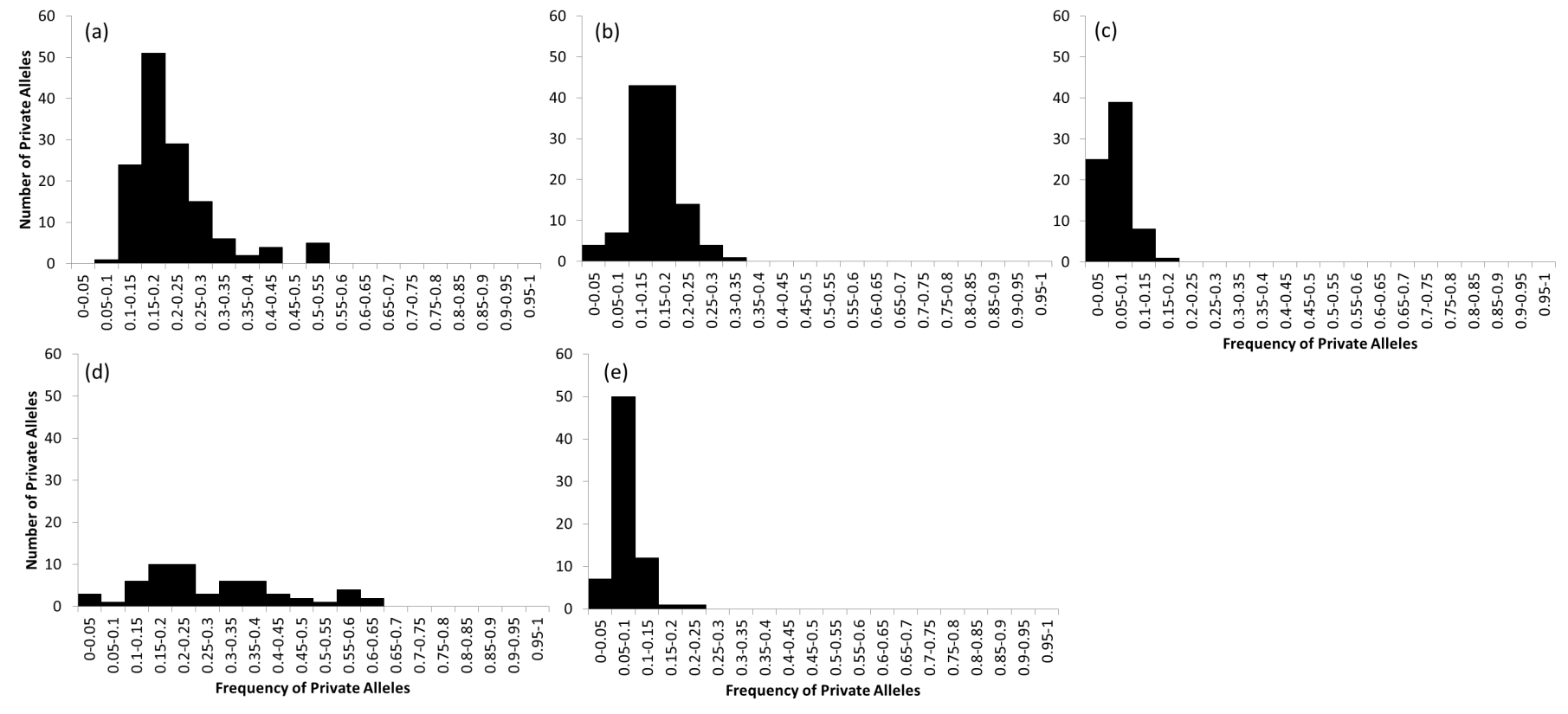


Figure S5 - % variance in stickleback morphology explained by PCA (top) and the association between PC1 and PC2 (bottom).


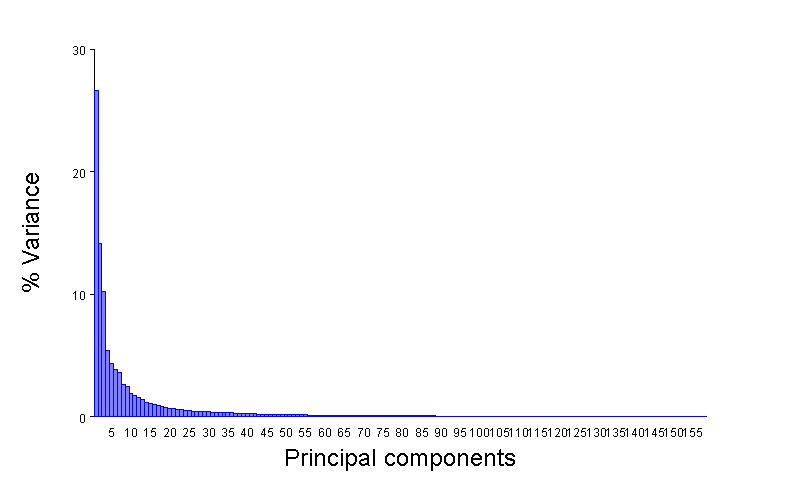


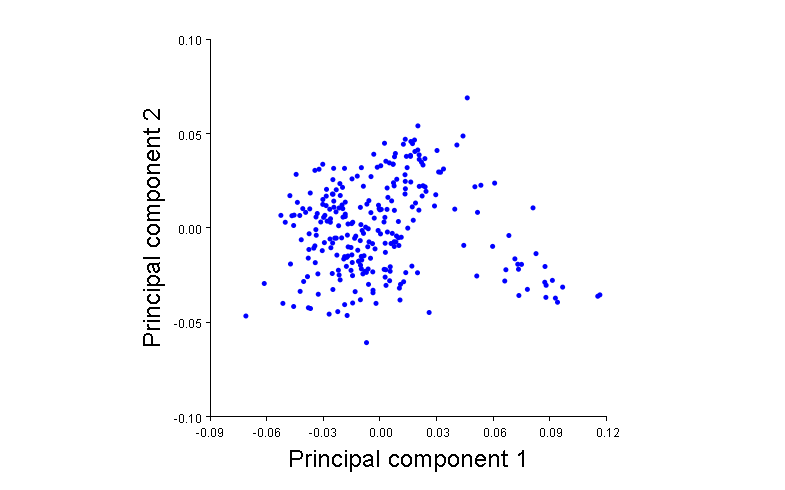


Figure S6 - Mantel tests for (a) geographic distance and neutral F_ST_, (b) geographic distance and plate P_ST_, (c) geographic distance and F_STQ_, (d) neutral F_ST_ and plate P_ST_, (e) neutral F_ST_ and F_STQ_, (f) Plate P_ST_ and F_STQ_. See Table 4 for associated statistics.


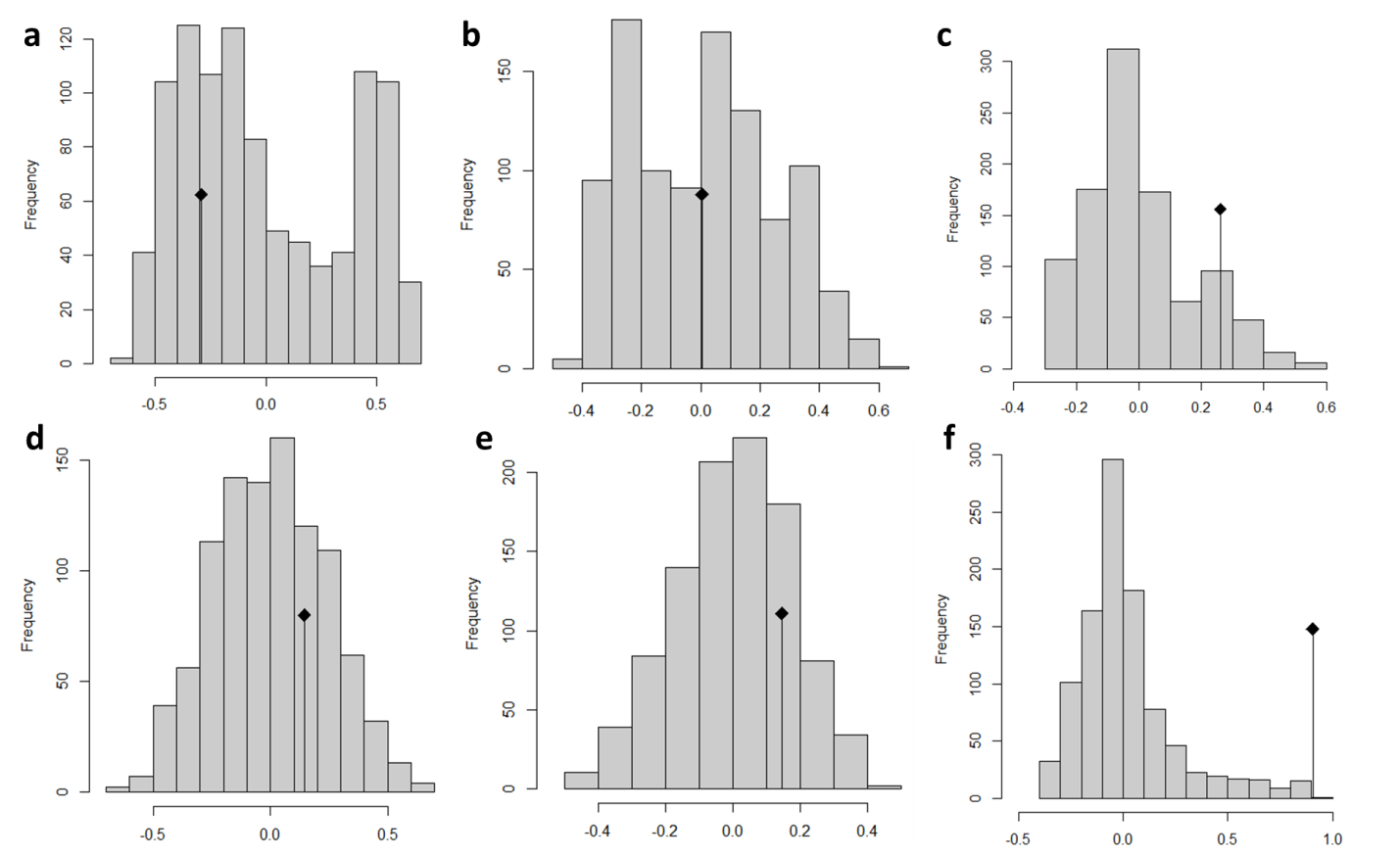


Figure S7 - Mantel tests for P_ST_ for PC1 (a-c), PC2 (d-f), PC3 (g-i), and PC4 (j-l) compared to geographic distance (a,d,g,j), neutral F_ST_ (b,e,h,k), and F_STQ_ (c,f,i,l). See Table 4 for associated statistics.


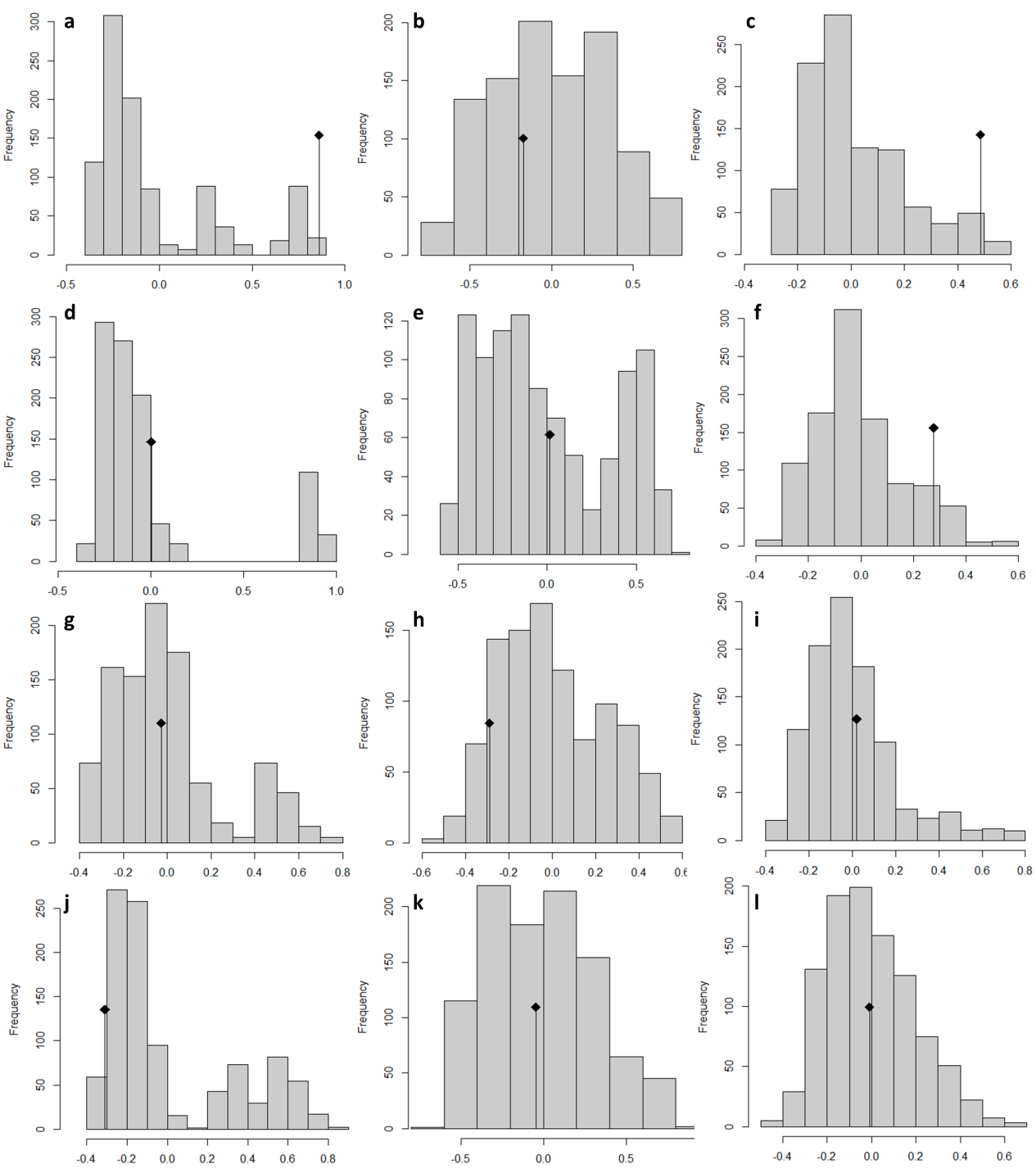


Figure S8 – Results of pseudo-observed data set (POD) for identifying the appropriate XtX threshold for the marine stickleback outlier analysis,


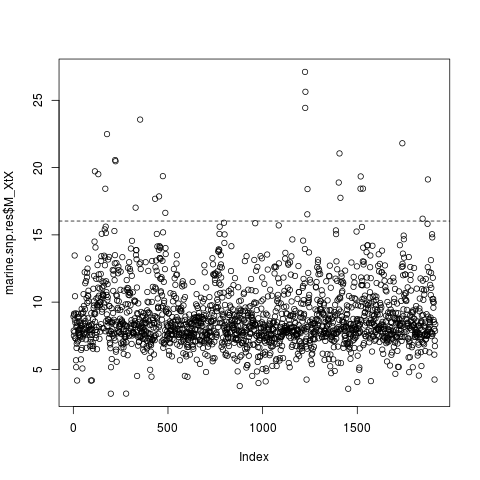

Supplement: Supplementary file 1 — Additional tables and figures. This document contains information supporting the main text, including: the list of landmarks used for 3D morphometrics, population-specific details on sex and platedness, additional information pertaining to methodology, the distributions of major allele frequencies and FIS per population, isolation-by-distance analyses, details regarding Adegenet-recognized clusters, CVA and DFA morphometrics results, additional Mantel tests, global pairwise FST values for each marine-freshwater comparison, and results pertaining to the outlier analysis. (DOCX 1008 kb) [file 12862_2018_1228_MOESM1_ESM.docx]
